# Attitudes, Perceptions, and Factors Influencing the Adoption of AI in Health Care Among Medical Staff: Nationwide Cross-Sectional Survey Study

**DOI:** 10.2196/75343

**Published:** 2025-08-08

**Authors:** Qianqian Dai, Ming Li, Maoshu Yang, Shiwu Shi, Zhaoyu Wang, Jiaojiao Liao, Zhaoji Li, Weinan E, Liyuan Tao, Yi-Da Tang

**Affiliations:** 1 Center for Data Science in Clinical Medicine, Peking University Third Hospital Beijing China; 2 Institute of Social Science Survey, Peking University Beijing China; 3 School of Basic Medicine, Peking University Beijing China; 4 Research Center of Clinical Epidemiology, Peking University Third Hospital Beijing China; 5 Center for Machine Learning Research and School of Mathematical Sciences, Peking University Beijing China; 6 Department of Cardiology and Institute of Vascular Medicine, Peking University Third Hospital Beijing China

**Keywords:** artificial intelligence, AI, medical staff, adoption, cross-sectional studies

## Abstract

**Background:**

Artificial intelligence (AI) has demonstrated transformative potential in the health care field; yet, its clinical adoption faces challenges such as inaccuracy, bias, and data privacy concerns. As the primary operators of AI systems, physicians and nurses play a pivotal role in integrating AI into clinical workflows. Their acceptance and use of AI are essential for bridging the gap between technological innovation and practical implementation. Exploring Chinese medical staff’s attitudes and identifying key factors influencing AI adoption are fundamental to developing targeted strategies to facilitate the effective application of AI in clinical settings.

**Objective:**

This study aims to investigate attitudes and perceptions regarding medical AI among physicians and nurses in China and identify the factors influencing its adoption.

**Methods:**

A nationwide cross-sectional survey was conducted online from December 12 to 26, 2024. Participants were recruited from the Chinese Medical Association and the Chinese Nursing Association. The structured questionnaire assessed demographic characteristics, knowledge and attitudes toward medical AI, experiences and insights regarding using medical AI, and perceptions and factors influencing the adoption of AI based on the unified theory of acceptance and use of technology (UTAUT) model. Multiple linear regression and Karlson-Holm-Breen mediation analysis were used to identify influencing factors. Sample weighting by regional distribution was applied for sensitivity analysis.

**Results:**

The survey included 991 physicians and 1714 nurses. Among the respondents, 92.4% (916/991) of the physicians and 84.19% (1443/1714) of the nurses reported awareness of medical AI applications, 22.8% (226/991) of the physicians and 17% (291/1714) of the nurses had used AI, and 82.6% (819/991) of the physicians and 80.22% (1375/1714) of the nurses held optimistic views about AI’s prospects. After adjusting for covariates, performance expectancy (physicians: B=0.144, 95% CI 0.092-0.197; nurses: B=0.292, 95% CI 0.245-0.338), effort expectancy (physicians: B=0.681, 95% CI 0.562-0.800; nurses: B=0.440, 95% CI 0.342-0.538), social influence (physicians: B=0.264, 95% CI 0.187-0.341; nurses: B=0.098, 95% CI 0.045-0.152), and facilitating conditions (physicians: B=0.098, 95% CI 0.030-0.165; nurses: B=0.158, 95% CI 0.105-0.212) had significant positive impacts on willingness to use AI. Perceived risk showed no significant effect on physicians’ intention to use AI (B=0.012, 95% CI −0.022 to 0.045) but negatively impacted nurses’ intention to use AI (B=−0.041, 95% CI −0.066 to −0.015). Performance expectancy and effort expectancy partially mediated the relationship between facilitating conditions and intention to use. Age, educational level, hospital level, work experience, and personal views also significantly influenced willingness. Weighted and unweighted analyses yielded consistent results, confirming the robustness of the findings.

**Conclusions:**

Substantial disparities exist between high willingness to adopt medical AI and its low actual use among Chinese medical staff. System optimization focusing on utility enhancement, workflow integration, and risk mitigation for medical staff, along with support for infrastructure and training, could accelerate AI adoption in clinical practice.

## Introduction

### Background

In recent years, artificial intelligence (AI) technology has experienced explosive growth in the medical field, emerging as a key driver of the transformation of the medical industry and presenting unprecedented opportunities and challenges to the traditional medical model [1]. AI technology has achieved remarkable results in health care, even surpassing human performance in some aspects. An increasing number of medical institutions have started to integrate AI technology into routine practice, including assistive diagnosis, clinical decision support, disease risk prediction, patient monitoring, and personal health assistants [2,3]. These applications of AI technology can not only improve the efficiency of clinical diagnosis and treatment, minimize human errors, and enhance the quality of medical services but also boost the productivity of medical workers, reduce their workload, and increase patient satisfaction [4]. Despite these advancements, the practical application of AI technology in clinical practice has not kept pace with scientific progress and continues to be associated with numerous risks, such as generating false content, providing inaccurate information, amplifying biases against underrepresented groups, and breaching personal privacy [5].

As the primary operators of medical AI systems, physicians and nurses play crucial roles in integrating AI tools into clinical workflows [6]. Consequently, comprehending the attitudes and intentions of medical staff toward the use of medical AI technology is of paramount importance. Recent studies indicate that health care practitioners generally hold optimistic views regarding the prospects of clinical AI [7]. However, awareness and rates of AI use in clinical practice remain low, and concerns regarding its accuracy, safety, ethics, and liability persist [8,9]. An international survey involving 2048 medical researchers revealed that 69.7% of the respondents were highly interested in AI chatbots, with 44.5% having used them [10]. In a survey of 284 colposcopists in China, >90% expressed a positive attitude toward the colposcopic AI auxiliary diagnostic system, and 53% reported awareness of it; yet, only 31% were familiar with its operation [11]. A survey conducted by the American Society for Gastrointestinal Endoscopy among 374 gastroenterologists found that 95.5% believed that AI solutions would positively impact clinical practice; however, only 6.7% had used AI in clinical settings, reflecting an overall positive outlook alongside concerns about technology and cost [12]. A survey of Australian mental health professionals indicated that 92% of the respondents believed that AI offered advantages in terms of accessibility, cost reduction, personalization, and work efficiency, but 51% expressed concerns about the risks of AI, including reduced interpersonal contact, ethical issues, privacy and regulatory concerns, medical errors, potential abuse, and data security [13]. These concerns among medical staff have, to some extent, impeded the widespread adoption of medical AI. Therefore, it is imperative to explore the underlying reasons for this gap to better align AI technology with clinical practice and to facilitate the effective application of AI in health care settings [14].

### The Unified Theory of Acceptance and Use of Technology

The implementation of AI in clinical practice is influenced by numerous interdependent factors. It is equally crucial to explore the factors associated with the attitudes and perceptions of medical staff toward the adoption of AI in health care. Previous studies have demonstrated that variations in views on AI can be ascribed to a range of factors, including age, sex, educational level, and experience related to AI [15]. Moreover, the technology acceptance model (TAM), the theory of planned behavior (TPB), and the unified theory of acceptance and use of technology (UTAUT) have offered theoretical frameworks for examining the acceptance of medical AI [16]. The UTAUT model integrates key factors from multiple traditional models applied to technology acceptance, aiming to understand and predict users’ intentions and behaviors related to the use of IT, with high universality and explanatory power [17]. The UTAUT model and its modified versions have been extensively used in research on the willingness to adopt technology in the health care sector [18-21].

The UTAUT model indicates that performance expectancy, effort expectancy, social influence, and facilitating conditions are the main predictors affecting users’ acceptance of new technologies. Performance expectancy refers to the degree to which an individual believes that using technology will improve their work performance. Effort expectancy involves the perception of the ease of using technology. Social influence refers to social factors, such as the expectations and attitudes of leaders and colleagues, that affect an individual’s intention to use technology. Facilitating condition represents the organizational and technical infrastructure required to adopt technology. Many studies have proven that these 4 predictors have a positive impact on users’ intention to use new technologies [18-21]. In addition, subsequent research has found that the impact of perceived risk on AI adoption intention cannot be ignored. Perceived risk refers to the potential negative consequences perceived when using new technology, defined by a combination of uncertainty and the seriousness of an outcome [22,23]. Different forms of perceived risk can reduce individuals’ intention to use new technology [24-26].

To better reflect the context of AI adoption in health care, we made some modifications to the original UTAUT model. First, we removed the “use behavior” component because most of the health care professionals (2188/2705, 80.89%) in our study had no prior experience using medical AI, making “intention to use” a more appropriate outcome variable [18,27,28]. Second, we added perceived risk to account for concerns specific to AI, such as misinformation, algorithmic bias, and data privacy issues, which have been shown to negatively affect technology adoption [5,24,29]. Third, we examined the mediating roles of performance expectancy and effort expectancy between facilitating conditions and intention to use, based on evidence suggesting that supportive infrastructure influences adoption through user confidence and perceived ease of use [30-32]. A visual representation of the revised model and hypotheses is provided in Multimedia Appendix 1.

### Objectives

Medical staff need to embrace the burgeoning potential of medical AI. Investigating health care workers’ willingness to use medical AI and the factors associated with it is essential. Through a nationwide online survey, this cross-sectional study aims to explore physicians’ and nurses’ knowledge and attitudes, their experience and insights related to use, and their perceptions of and intention to use medical AI. On the basis of the UTAUT model, this study explores the impact of performance expectancy, effort expectancy, social influence, facilitating conditions, and perceived risk on physicians’ and nurses’ intention to use medical AI. It aims to understand the actual needs of medical staff, identify the challenges they face in accepting new technologies, and develop feasible strategies to better support the effective use of medical AI to improve patient services in clinical practice.

## Methods

### Study Design and Participant Recruitment

This study used a nationwide cross-sectional survey design, targeting physicians and nurses from various regions in China. The survey was conducted from December 12 to December 26, 2024. A nonrandom, convenience sampling method was used to recruit participants. To enhance the representativeness of the sample, we partnered with authoritative institutions. Specifically, the survey was developed using Wenjuanxing [33] (a widely used online survey platform in China), and a unique QR code was generated for dissemination. With the support of the Chinese Medical Association and the Chinese Nursing Association, which represent physicians and nurses, respectively, in China’s health care system, we contacted their provincial branches. These provincial associations then engaged hospital administrators, who distributed the survey link via WeChat groups (a feature of WeChat [Tencent], a widely used communication platform in China) for health care workers to complete anonymously and voluntarily. To improve regional representation, we proactively contacted the responsible personnel at hospitals with low response rates during the data collection period to encourage participation and ensure a more balanced geographic distribution of respondents.

The introduction to the survey clearly explained its purpose. To prevent careless responses associated with short response times, we excluded participants who spent <180 seconds on the survey, thereby enhancing the accuracy and reliability of the data. The study adhered to the STROBE (Strengthening the Reporting of Observational Studies in Epidemiology) reporting guideline.

### Ethical Considerations

This study was approved by the ethics review committee of Peking University Third Hospital (M20241008) and was conducted in accordance with the ethical guidelines of the Declaration of Helsinki. Informed consent was obtained from participants before they answered the survey questions. All collected data were kept strictly confidential and anonymous. Participation was voluntary, and no incentives were provided for survey completion.

### Questionnaire Information

After the initial development of the questionnaire, we sought expert input from 3 professionals: a physician, a nurse, and an epidemiologist. Their feedback was instrumental in refining ambiguous or inappropriate items. In particular, they recommended adding an introductory section on the scope and examples of AI applications in health care to provide respondents with a clearer understanding of this emerging technology. Considering that AI adoption in clinical practice is still at an early stage and that many health care professionals may lack hands-on experience with AI tools, the experts also advised us to focus primarily on intention to use rather than actual use behavior. On the basis of the revised version, we conducted a small-scale pilot survey with 30 physicians and 34 nurses to assess the reliability of the instrument. The results demonstrated high internal consistency across all constructs. Specifically, the Cronbach α values were 0.948 for performance expectancy, 0.968 for effort expectancy, 0.912 for social influence, 0.962 for facilitating conditions, 0.932 for perceived risk, and 0.964 for intention to use, indicating good reliability across all constructs.

The final questionnaire was structured into 4 sections: demographic characteristics, knowledge and attitudes toward AI in health care, experiences and insights regarding using medical AI, and perceptions and factors influencing the adoption of AI in health care.

Demographic characteristics included age, sex, province, educational level, occupation, hospital level, department, title, and work experience. These details were incorporated to provide a comprehensive description and analysis of the sample, establishing a basis for exploring differences in views and intention to use medical AI among health care workers with varying characteristics.

Knowledge and attitudes toward AI were assessed through questions about participants’ awareness of medical AI, their experience using it, attention to medical AI in the workplace, personal views on AI’s prospects, and interest in AI applications. These questions aimed to assess health care professionals’ overall knowledge and attitudes toward medical AI, laying the groundwork for a more in-depth analysis of the factors influencing their intention to use AI in clinical practice.

Experiences and insights regarding using AI were further explored among physicians and nurses who had used AI in health care. This section included questions about the duration of use, satisfaction with AI, the most frequently used functions, and areas requiring urgent improvement. These questions sought to provide a deep understanding of health care professionals’ practical experiences and perceptions of using medical AI. The insights gathered offer valuable feedback for optimizing medical AI products and services and provide empirical data to analyze the relationship between experience using AI and the intention to continue using AI.

Perceptions and factors influencing the adoption of medical AI formed the core content of the questionnaire and were assessed using a 5-point Likert scale. This section included 6 dimensions: performance expectancy, effort expectancy, social influence, facilitating conditions, perceived risk, and intention to use. Each item was scored according to the following response options: 1=strongly disagree, 2=disagree, 3=neutral, 4=agree, and 5=strongly agree. Performance expectancy evaluated the degree to which medical staff believed that using medical AI could improve work performance; for example, “I believe that using medical AI can improve my work efficiency,” with higher scores indicating higher performance expectancy. Effort expectancy measured the perceived effort required by health care workers to use medical AI; for example, “I believe I can easily master the use of medical AI,” with higher scores indicating greater ease of use. Social influence reflected social factors, such as the expectations and attitudes of leaders and colleagues, that affect the intention of health care professionals to use medical AI; for example, “My colleagues are actively using medical AI,” with higher scores indicating more positive social influence. Facilitating conditions examined the support provided by medical institutions to enable health care professionals to use medical AI; for example, “The hospital can provide me with sufficient medical AI equipment,” with higher scores indicating greater facilitating conditions. Perceived risk assessed the degree to which health care professionals were concerned about the potential risks of using medical AI; for example, “I am worried that medical AI will provide me with incorrect information,” with higher scores indicating greater perceived risk. Intention to use directly assessed health care professionals’ future willingness to use medical AI; for example, “I am willing to use medical AI to assist me in my work,” with higher scores indicating stronger intention to use. The specific questionnaire items are provided in Multimedia Appendix 2.

During the formal survey phase, confirmatory factor analysis was conducted to evaluate whether the collected data adequately supported the measurement items for each construct in the modified UTAUT model. Reliability was assessed using Cronbach α and composite reliability, while validity was evaluated through convergent and discriminant validity. The results indicated that the questionnaire met recommended psychometric standards for both reliability and validity (Multimedia Appendices 3 and 4). In addition, model fit was evaluated using common indices. The root mean square error of approximation was 0.062, the comparative fit index was 0.966, the Tucker-Lewis index was 0.960, and the standardized root mean square residual was 0.040—all within acceptable thresholds, indicating good model fit. In summary, the questionnaire demonstrated strong reliability and validity, providing a robust basis for subsequent analyses.

### Statistical Analysis

First, descriptive statistical analysis was conducted to summarize the demographic characteristics of all participants. Frequencies and percentages were calculated for categorical variables to describe the sample composition, while continuous variables were presented as means and SDs. In addition, the distribution of responses concerning AI perceptions was analyzed by reporting means, SDs, and the proportion of agreement, wherein responses of “strongly agree” or “agree” were categorized as “agree.” This approach facilitated a preliminary understanding of the overall trends in medical staff perceptions across different dimensions, serving as a crucial reference for subsequent in-depth analyses.

Second, multiple linear regression models were used to examine the relationships between demographic characteristics, AI knowledge, AI use, perceived hospital attention to medical AI, personal views on AI’s prospects, and intention to use AI among physicians and nurses. After adjusting for potential confounding factors, the analysis further explored the associations between performance expectancy, effort expectancy, social influence, facilitating conditions, perceived risk, and intention to use. Regression coefficients (B) and 95% CIs were calculated to assess the impact of each independent variable on the dependent variable. This analysis identified significant factors influencing health care professionals’ intention to use medical AI, guiding the formulation of targeted intervention measures.

Third, a mediation analysis was conducted to explore the mediating roles of performance expectancy and effort expectancy between facilitating conditions and intention to use. Using the Karlson-Holm-Breen method, the total, direct, and indirect effects of facilitating conditions on intention to use were examined, along with the magnitude of the mediating effects [34,35]. This approach uncovered potential psychological mechanisms underlying the formation of physicians’ and nurses’ intention to use medical AI.

Fourth, health care professionals from different clinical departments may have different levels of exposure to and demand for medical AI technologies. This can in turn influence their perceptions and behavioral intentions. Similarly, prior familiarity with medical AI may also shape individuals’ attitudes and intentions regarding its use. Therefore, we conducted subgroup analyses to examine differences in intention to use medical AI across clinical departments and based on participants’ prior awareness of medical AI.

Finally, a sensitivity analysis was performed to test the robustness of the results by weighting samples based on the stratified distribution of physicians and nurses across different regions as reported in the China Statistical Yearbook 2024 and reanalyzing the impact of various factors on intention to use AI [36]. This step confirmed the reliability and generalizability of the research conclusions. All statistical analyses were performed using Stata (version 18.0; StataCorp LLC), with a 2-tailed *P*<.05 considered statistically significant.

## Results

### Basic Characteristics of Participants

During the formal investigation phase, 3131 completed questionnaires were collected. Of these 3131 responses, 194 (6.2%) submitted by individuals whose occupations were not classified as physicians or nurses (eg, pharmacists, administrative staff, and researchers) and 232 (7.41%) that were completed in <180 seconds were excluded, leaving 2705 (86.39%) valid responses for analysis.

The 2705 respondents included 991 physicians (36.64%) and 1714 nurses (63.36%). As shown in Table 1, of the 991 physicians, 457 (46.1%) were male, and 534 (53.9%) were female. Of the 1714 nurses, 244 (14.24%) were male, and 1470 (85.76%) were female. The average age of the physicians was 40.1 (SD 9.3) years, while that of the nurses was 36.7 (SD 8.3) years. The sample was distributed across 6 geographic regions, with the north China region having the highest number of participants overall (844/2705, 31.2%). Of the 991 physicians, 568 (57.3%) held a master’s degree or higher, 862 (87%) were from tertiary hospitals, 609 (61.5%) worked in internal medicine departments, 441 (44.5%) held senior titles, and 285 (28.8%) had ≥21 years of work experience. Of the 1714 nurses, 1381 (80.57%) held a bachelor’s degree (n=44, 2.57% held a master’s degree or higher), 1299 (75.79%) worked at tertiary hospitals, 976 (56.94%) worked in internal medicine departments, 302 (17.62%) held senior titles, and 433 (25.26%) had ≥21 years of work experience.

**Table 1:** Demographics of participants.

Characteristics		Total (n=2705)	Physicians (n=991)	Nurses (n=1714)	*P* value	
**Sex, n (%)**						<.001
	Male	701 (25.9)	457 (46.1)	244 (14.2)		
	Female	2004 (74.1)	534 (53.9)	1470 (85.8)		
Age (overall; y), mean (SD)		38.0 (8.8)	40.1 (9.3)	36.7 (8.3)	<.001	
**Age (y), n (%)**						<.05
	<30	474 (17.5)	125 (12.6)	349 (20.4)		
	30-44	1607 (59.4)	560 (56.5)	1047 (61.1)		
	≥45	624 (23.1)	306 (30.9)	318 (18.6)		
**Region, n (%)**						<.001
	North China	844 (31.2)	256 (25.8)	588 (34.3)		
	Northeast China	290 (10.7)	119 (12)	171 (10)		
	East China	736 (27.2)	258 (26)	478 (27.9)		
	Central south China	431 (15.9)	234 (23.6)	197 (11.5)		
	Southwest China	244 (9)	53 (5.3)	191 (11.1)		
	Northwest China	160 (5.9)	71 (7.2)	89 (5.2)		
**Educational level, n (%)**						<.001
	Associate’s degree or lower	310 (11.5)	21 (2.1)	289 (16.9)		
	Bachelor’s degree	1783 (65.9)	402 (40.6)	1381 (80.6)		
	Master’s degree or higher	612 (22.6)	568 (57.3)	44 (2.6)		
**Hospital level, n (%)**						<.001
	Tertiary hospital	2161 (79.9)	862 (87)	1299 (75.8)		
	Secondary hospital or lower	544 (20.1)	129 (13)	415 (24.2)		
**Department, n (%)**						<.001
	Internal medicine	1585 (58.6)	609 (61.5)	976 (56.9)		
	Surgery	517 (19.1)	220 (22.2)	297 (17.3)		
	Medical technology	356 (13.2)	88 (8.9)	268 (15.6)		
	Other	247 (9.1)	74 (7.5)	173 (10.1)		
**Professional title, n (%)**						<.001
	Senior	743 (27.5)	441 (44.5)	302 (17.6)		
	Intermediate	1110 (41)	338 (34.1)	772 (45)		
	Junior	792 (29.3)	185 (18.7)	607 (35.4)		
	None	60 (2.2)	27 (2.7)	33 (1.9)		
**Work experience (y), n (%)**						<.001
	≤10	954 (35.3)	382 (38.5)	572 (33.4)		
	11-20	1033 (38.2)	324 (32.7)	709 (41.4)		
	≥21	718 (26.5)	285 (28.8)	433 (25.3)		

### Knowledge and Attitudes Toward Medical AI

Table 2 presents the descriptive analysis results regarding participants’ knowledge and attitudes toward medical AI. Of the 991 physicians, 916 (92.4%) had heard of medical AI, but only 226 (22.8%) had actually used it in their work; in addition, 710 (71.6%) reported that their hospitals paid general or high attention to AI in health care, and 819 (82.6%) were optimistic about the prospects of medical AI. Of the 1714 nurses, 1443 (84.19%) had heard of medical AI, but only 291 (17%) had used it in their work; furthermore, 1239 (72.29%) indicated that their hospitals paid general or high attention to medical AI, and 1375 (80.22%) were optimistic about the prospects of medical AI.

**Table 2:** Knowledge and attitudes toward medical artificial intelligence (AI) of participants.

Items		Total (n=2705), n (%)	Physicians (n=991), n (%)	Nurses (n=1714), n (%)	*P* value	
**Have you ever heard of medical AI?**						<.001
	No	346 (12.8)	75 (7.6)	271 (15.8)		
	Yes	2359 (87.2)	916 (92.4)	1443 (84.2)		
**Have you ever used medical AI?**						<.001
	No	2188 (80.9)	765 (77.2)	1423 (83)		
	Yes	517 (19.1)	226 (22.8)	291 (17)		
**What is the level of attention your hospital pays to medical AI?**						.68
	Low	756 (27.9)	281 (28.4)	475 (27.7)		
	General	1080 (39.9)	385 (38.8)	695 (40.5)		
	High	869 (32.1)	325 (32.8)	544 (31.7)		
**What is your view on the prospects of medical AI?**						.12
	Pessimistic	511 (18.9)	172 (17.4)	339 (19.8)		
	Optimistic	2194 (81.1)	819 (82.6)	1375 (80.2)		

The top 3 applications of interest to physicians were assistive diagnosis (841/991, 84.9%), medical data management (788/991, 79.5%), and teaching and research (700/991, 70.6%). By contrast, the nurses’ top 3 areas of interest were patient monitoring (1409/1714, 82.21%), medical data management (1223/1714, 71.35%), and disease surveillance (1192/1714, 69.54%). These differences reflect the distinct responsibilities and focus of physicians and nurses in medical work and provide targeted references for the application of medical AI in different medical scenarios.

### Experiences and Insights Regarding Using Medical AI

Of the 226 physicians who had used medical AI, 168 (74.3%) had used it for <2 years. Of the 226 physicians, 132 (58.4%) expressed satisfaction with their experience using medical AI, whereas only 9 (4%) reported dissatisfaction. The 3 most common applications of medical AI among the physicians were assistive diagnosis (159/226, 70.4%), teaching and research (129/226, 57.1%), and medical data management (128/226, 56.6%). Regarding the 291 nurses who had used medical AI, 185 (63.6%) had used it for <2 years. Notably, of the 291 nurses, 197 (67.7%) were satisfied with their experience using medical AI, while only 2 (0.7%) expressed dissatisfaction. The top 3 applications of medical AI among the nurses were patient monitoring (194/291, 66.7%), medical data management (179/291, 61.5%), and disease surveillance (159/291, 54.6%). In terms of suggestions for improvement, both physicians and nurses highlighted the urgent necessity to enhance system stability, reliability, and usability; strengthen data security and privacy protection; and improve system response speed and processing capabilities. These enhancements are of paramount importance for increasing the intention of medical staff to adopt AI in health care settings.

### Perceptions of and Intention to Use Medical AI

The 6 aforementioned dimensions were quantitatively measured through 2 to 5 questions each (Table 3). In terms of performance expectancy, 76.3% (756/991) to 86.5% (857/991) of the physicians and 71.76% (1230/1714) to 85.65% (1468/1714) of the nurses recognized the positive impact of medical AI on their work, indicating that the majority of health care workers in China hold a positive attitude toward the value of AI. Regarding effort expectancy, 85% (842/991) to 88.4% (876/991) of the physicians and 83.37% (1429/1714) to 88.39% (1515/1714) of the nurses believed that they could master the use of AI, reflecting the confidence of medical staff in their ability to adapt to new technology. With regard to social influence, 47.5% (471/991) to 52.9% (524/991) of the physicians and 45.51% (780/1714) to 49.94% (856/1714) of the nurses felt that the people around them had a positive impact on their use of AI. In terms of facilitating conditions, 46.4% (460/991) to 50.6% (501/991) of the physicians and 46.67% (800/1714) to 53.03% (909/1714) of the nurses believed that their medical institutions could provide resource support, such as technical training and equipment provision, ensuring the application of AI in health care.

**Table 3:** Participants’ perceptions of and intention to use medical artificial intelligence (AI).

Dimensions and items		Total (n=2705)		Physicians (n=991)		Nurses (n=1714)	
		Score, mean (SD)	Agree, n (%)^a^	Score, mean (SD)	Agree, n (%)^a^	Score, mean (SD)	Agree, n (%)^a^
**Performance Expectancy**							
	1	4.02 (0.84)	2006 (74.2)	4.05 (0.86)	776 (78.3)	4.00 (0.82)	1230 (71.8)
	2	4.23 (0.76)	2325 (86)	4.22 (0.80)	857 (86.5)	4.24 (0.73)	1468 (85.6)
	3	4.14 (0.81)	2184 (80.7)	4.10 (0.85)	796 (80.3)	4.16 (0.78)	1388 (81)
	4	4.10 (0.82)	2103 (77.7)	4.04 (0.88)	756 (76.3)	4.13 (0.79)	1347 (78.6)
**Effort Expectancy**							
	1	4.14 (0.74)	2271 (84)	4.15 (0.75)	842 (85)	4.14 (0.73)	1429 (83.4)
	2	4.21 (0.67)	2391 (88.4)	4.19 (0.70)	876 (88.4)	4.22 (0.65)	1515 (88.4)
**Social Influence**							
	1	3.53 (0.92)	1380 (51)	3.56 (0.89)	524 (52.9)	3.52 (0.93)	856 (49.9)
	2	3.49 (0.90)	1259 (46.5)	3.49 (0.88)	471 (47.5)	3.48 (0.90)	788 (46)
	3	3.51 (0.86)	1255 (46.4)	3.51 (0.88)	475 (47.9)	3.51 (0.85)	780 (45.5)
**Facilitating Conditions**							
	1	3.48 (0.91)	1260 (46.6)	3.44 (0.93)	460 (46.4)	3.50 (0.90)	800 (46.7)
	2	3.56 (0.89)	1410 (52.1)	3.49 (0.91)	501 (50.6)	3.60 (0.88)	909 (53)
	3	3.54 (0.85)	1341 (49.6)	3.47 (0.89)	481 (48.5)	3.58 (0.83)	860 (50.2)
**Perceived Risk**							
	1	3.39 (0.82)	1181 (43.7)	3.44 (0.82)	469 (47.3)	3.36 (0.82)	712 (41.5)
	2	3.47 (0.83)	1320 (48.8)	3.54 (0.84)	526 (53.1)	3.42 (0.83)	794 (46.3)
	3	3.38 (0.84)	1178 (43.5)	3.44 (0.83)	466 (47)	3.34 (0.84)	712 (41.5)
	4	3.18 (0.87)	889 (32.9)	3.23 (0.89)	350 (35.3)	3.15 (0.87)	539 (31.4)
	5	3.04 (0.94)	762 (28.2)	3.00 (0.96)	277 (28)	3.07 (0.92)	485 (28.3)
**Intention to Use**							
	1	3.99 (0.68)	2114 (78.2)	3.99 (0.70)	783 (79)	3.99 (0.67)	1331 (77.7)
	2	3.94 (0.71)	2011 (74.3)	3.94 (0.73)	747 (75.4)	3.94 (0.71)	1264 (73.7)
	3	3.92 (0.72)	1968 (72.8)	3.89 (0.75)	714 (72)	3.93 (0.71)	1254 (73.2)
	4	4.05 (0.69)	2174 (80.4)	4.05 (0.70)	804 (81.1)	4.05 (0.68)	1370 (79.9)

^a^Perceptions of medical AI were assessed using a 5-point Likert scale; responses of “strongly agree” or “agree” were categorized as “agree.”

Regarding the perception of risks associated with AI use, 53.1% (526/991) of the physicians and 46.32% (794/1714) of the nurses expressed concerns about the potential for medical disputes and difficulties in determining liability when using medical AI. In addition, 47.3% (469/991) of the physicians and 41.54% (712/1714) of the nurses worried that medical AI might provide incorrect information, leading to adverse consequences. Furthermore, 47% (466/991) of the physicians and 41.54% (712/1714) of the nurses had doubts about the transparency and explainability of medical AI, while 35.3% (350/991) of the physicians and 31.44% (539/1714) of the nurses were concerned that medical AI would exacerbate the unfairness of medical services. Relatively speaking, only 28.17% (762/2705) of the health care workers worried that their jobs would be replaced by medical AI in the future. Ultimately, 72% (714/991) to 81.1% (804/991) of the physicians and 73.16% (1254/1714) to 79.93% (1370/1714) of the nurses expressed their intention to use medical AI, highlighting the open attitude and strong interest of health care professionals in the future integration of medical AI into their daily work. This indicates that despite the numerous risks associated with medical AI in clinical practice, there remains considerable potential for its widespread adoption and promotion.

### Factors Influencing the Adoption of AI in Health Care

**Figure 1:**
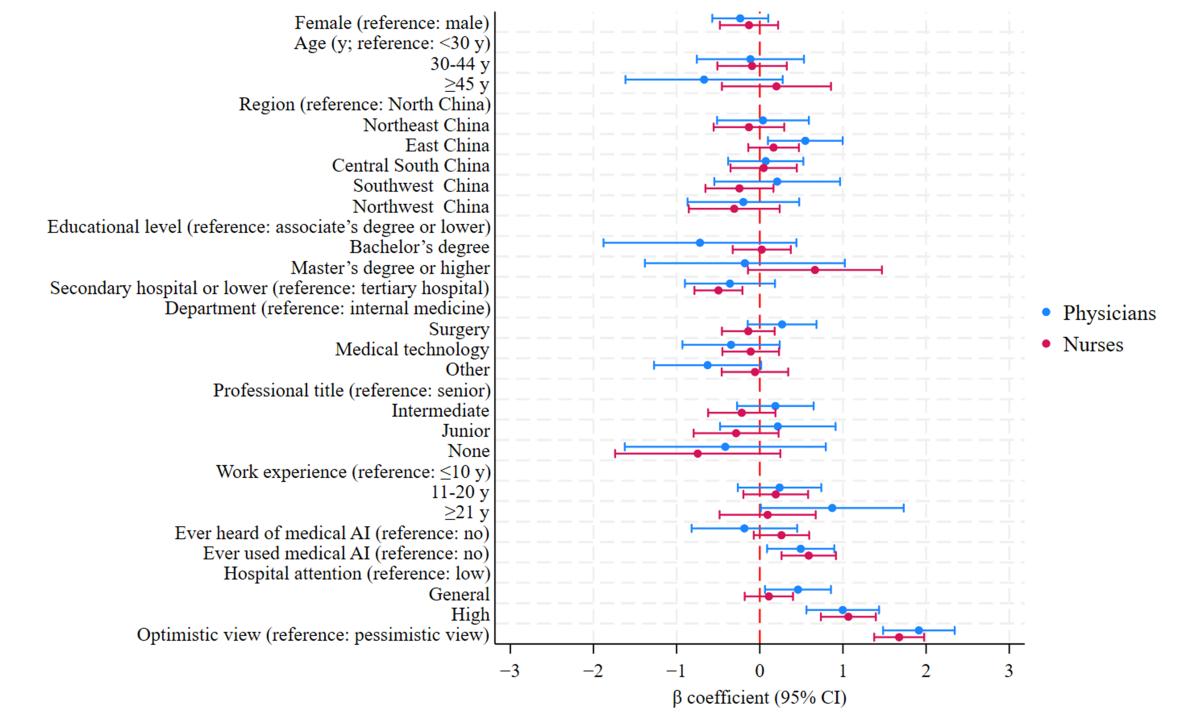
Factors associated with intention to use medical artificial intelligence (AI).

We conducted a multiple linear regression analysis to identify factors associated with intention to use medical AI, and the results are presented in Figure 1. For the physicians, significant positive predictors included being from the east China region (B=0.54, 95% CI 0.098-0.997; *P*=.02), having >20 years of work experience (B=0.872, 95% CI 0.013-1.731; *P*=.047), having experience using medical AI (B=0.492, 95% CI 0.088-0.896; *P*=.02), working in a hospital with high attention to medical AI (B=0.998, 95% CI 0.561-1.435; *P*<.001), and having an optimistic view on the prospects of medical AI (B=1.914, 95% CI 1.483-2.344; *P*<.001). For the nurses, working in a secondary hospital or lower (B=−0.497, 95% CI −0.786 to −0.208; *P*=.001) had a significant negative impact on intention to use AI. Having experience using medical AI (B=0.589, 95% CI 0.262-0.916; *P*<.001), working in a hospital with high attention to medical AI (B=1.066, 95% CI 0.735-1.396; *P*<.001), and having an optimistic view on the prospects of medical AI (B=1.676, 95% CI 1.376-1.977; *P*<.001) had significant positive impacts on intention to use AI (Multimedia Appendix 5).

**Figure 2:**
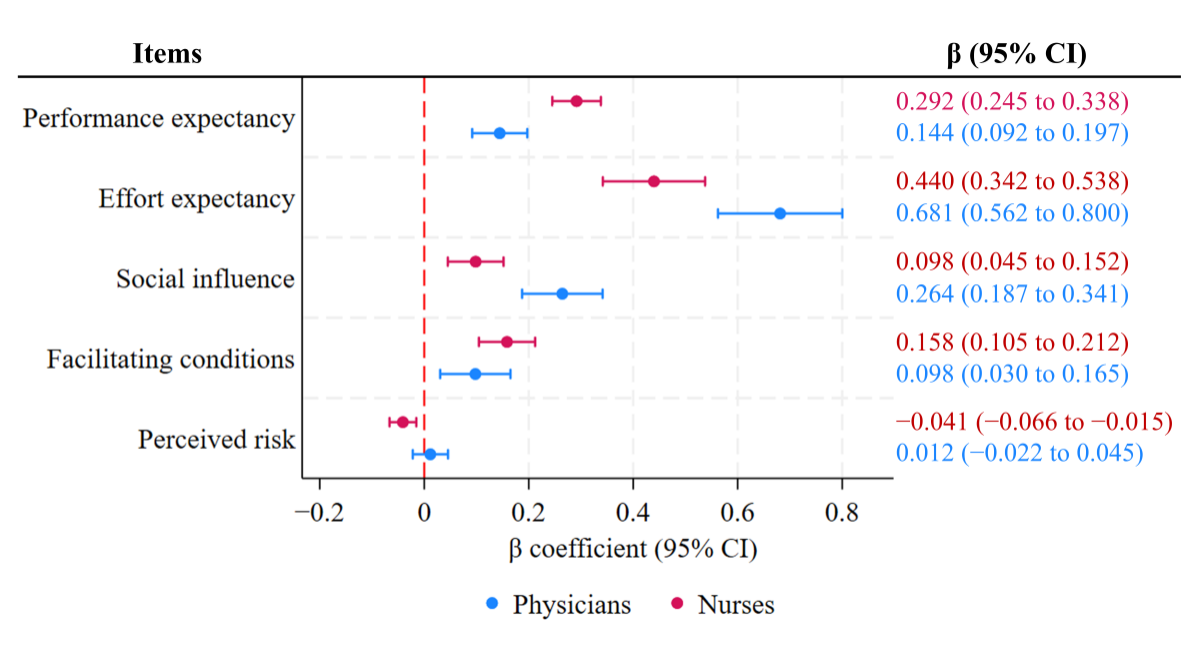
Relationship between perception of and intention to use medical artificial intelligence (AI). The multiple linear regression analysis was adjusted for demographic information, knowledge of AI, experience of using AI, hospital attention to AI, and personal views on the prospects of medical AI.

After further adjusting for participants’ demographic information, knowledge of AI, experience using AI, hospital attention to medical AI, and personal views on the prospects of medical AI as covariates (Table 4; Figure 2), performance expectancy (B=0.144, 95% CI 0.092-0.197; *P*<.001), effort expectancy (B=0.681, 95% CI 0.562-0.800; *P*<.001), social influence (B=0.264, 95% CI 0.187-0.341; *P*<.001), and facilitating conditions (B=0.098, 95% CI 0.030-0.165; *P*=.004) had significant positive impacts on the physicians’ intention to use medical AI, while perceived risk had no significant impact (B=0.012, 95% CI −0.022 to 0.045; *P*=.50). In addition, physicians aged ≥45 years (B=−0.788, 95% CI −1.499 to −0.077; *P*=.03) and those from a secondary hospital or lower (B=−0.421, 95% CI −0.827 to −0.015; *P*=.04) had weaker intention to use medical AI. By contrast, physicians with ≥21 years of work experience (B=0.712, 95% CI 0.067-1.357; *P*=.03) and those with positive views on the prospects of AI (B=0.548, 95% CI 0.207-0.889; *P*=.002) had stronger intention to use medical AI.

**Table 4:** Multiple linear regression model for estimating physicians’ and nurses’ intention to use medical artificial intelligence (AI).

Items		Total (n=2705)			Physicians (n=991)			Nurses (n=1714)	
		B (95% CI)	*P* value	B (95% CI)		*P* value	B (95% CI)		*P* value
**Dimensions**									
	Performance expectancy	0.226 (0.191 to 0.261)	*<.001* ^a^	0.144 (0.092 to 0.197)		*<.001*	0.292 (0.245 to 0.338)		*<.001*
	Effort expectancy	0.550 (0.474 to 0.626)	*<.001*	0.681 (0.562 to 0.800)		*<.001*	0.440 (0.342 to 0.538)		*<.001*
	Social influence	0.156 (0.112 to 0.200)	*<.001*	0.264 (0.187 to 0.341)		*<.001*	0.098 (0.045 to 0.152)		*<.001*
	Facilitating conditions	0.137 (0.095 to 0.179)	*<.001*	0.098 (0.030 to 0.165)		*.004*	0.158 (0.105 to 0.212)		*<.001*
	Perceived risk	−0.018 (−0.038 to 0.003)	.09	0.012 (−0.022 to 0.045)		.50	−0.041 (−0.066 to −0.015)		*.002*
**Sex (reference: male)**									
	Female	−0.115 (−0.297 to 0.066)	.21	−0.143 (−0.396 to 0.109)		.27	−0.016 (−0.296 to 0.264)		.91
**Age (y; reference: <30 y)**									
	30-44	−0.056 (−0.323 to 0.212)	.68	−0.037 (−0.522 to 0.447)		.88	−0.151 (−0.484 to 0.182)		.37
	≥45	0.032 (−0.384 to 0.448)	.88	−0.788 (−1.499 to −0.077)		*.03*	0.344 (−0.178 to 0.866)		.20
**Region (reference: north China)**									
	Northeast China	−0.099 (−0.360 to 0.163)	.46	−0.005 (−0.419 to 0.409)		.98	−0.118 (−0.457 to 0.220)		.49
	East China	0.044 (−0.153 to 0.240)	.67	0.185 (−0.155 to 0.524)		.29	−0.007 (−0.250 to 0.235)		.95
	Central south China	0.016 (−0.214 to 0.246)	.89	−0.067 (−0.407 to 0.272)		.70	0.105 (−0.213 to 0.423)		.52
	Southwest China	−0.001 (−0.282 to 0.279)	.99	−0.030 (−0.598 to 0.537)		.92	0.028 (−0.299 to 0.354)		.87
	Northwest China	−0.298 (−0.628 to 0.032)	.08	−0.452 (−0.958 to 0.054)		.08	−0.159 (−0.594 to 0.276)		.47
**Educational level (reference: associate’s degree or lower)**									
	Bachelor’s degree	0.124 (−0.132 to 0.381)	.34	−0.480 (−1.350 to 0.390)		.28	0.129 (−0.149 to 0.407)		.36
	Master’s degree or higher	0.364 (0.017 to 0.712)	*.04*	−0.460 (−1.362 to 0.441)		.32	0.942 (0.299 to 1.585)		*.004*
**Hospital level (reference: tertiary hospital)**									
	Secondary hospital or lower	−0.282 (−0.480 to −0.084)	*.005*	−0.421 (−0.827 to −0.015)		*.04*	−0.273 (−0.504 to −0.043)		*.02*
**Department (reference: internal medicine)**									
	Surgery	0.045 (−0.149 to 0.239)	.65	0.145 (−0.166 to 0.455)		.36	−0.024 (−0.277 to 0.229)		.85
	Medical technology	−0.143 (−0.372 to 0.085)	.22	−0.341 (−0.781 to 0.098)		.13	−0.127 (−0.399 to 0.144)		.36
	Other	0.000 (−0.265 to 0.265)	.99	−0.269 (−0.753 to 0.214)		.28	0.122 (−0.196 to 0.441)		.45
**Professional title (reference: senior)**									
	Intermediate	−0.153 (−0.381 to 0.074)	.19	−0.125 (−0.471 to 0.222)		.48	−0.224 (−0.547 to 0.100)		.18
	Junior	−0.314 (−0.624 to −0.003)	*.048*	−0.245 (−0.771 to 0.280)		.36	−0.395 (−0.805 to 0.014)		.06
	None	−0.355 (−0.940 to 0.229)	.23	−0.675 (−1.581 to 0.232)		.14	−0.295 (−1.089 to 0.499)		.47
**Work experience (y; reference: ≤10 y)**									
	11-20	0.069 (−0.163 to 0.301)	.56	0.024 (−0.353 to 0.402)		.90	0.179 (−0.131 to 0.489)		.26
	≥21	0.053 (−0.317 to 0.424)	.78	0.712 (0.067 to 1.357)		*.03*	−0.217 (−0.679 to 0.244)		.36
**Ever heard of medical AI (reference: no)**									
	Yes	−0.002 (−0.234 to 0.229)	.98	−0.248 (−0.724 to 0.229)		.31	0.078 (−0.188 to 0.344)		.56
**Ever used medical AI (reference: no)**									
	Yes	0.096 (−0.106 to 0.298)	.35	0.227 (−0.083 to 0.537)		.15	0.048 (−0.219 to 0.315)		.73
**Hospital attention to medical AI (reference: low)**									
	General	−0.009 (−0.194 to 0.175)	.92	0.135 (−0.166 to 0.436)		.38	−0.105 (−0.337 to 0.127)		.38
	High	0.151 (−0.062 to 0.364)	.16	0.025 (−0.316 to 0.367)		.88	0.216 (−0.055 to 0.487)		.12
**View on prospects of medical AI (reference: pessimistic)**									
	Optimistic	0.472 (0.268 to 0.676)	*<.001*	0.548 (0.207 to 0.889)		*.002*	0.390 (0.136 to 0.644)		*.003*
**Occupation (reference:** **physician** **)**									
	Nurse	0.273 (0.055 to 0.491)	*.01*	N/A^b^		N/A	N/A		N/A

^a^Italicization indicates values that met the significance threshold.

^b^N/A: not applicable.

For the nurses, after adjusting for covariates, performance expectancy (B=0.292, 95% CI 0.245-0.338; *P*<.001), effort expectancy (B=0.440, 95% CI 0.342-0.538; *P*<.001), social influence (B=0.098, 95% CI 0.045-0.152; *P*<.001), and facilitating conditions (B=0.158, 95% CI 0.105-0.212; *P*<.001) had significant positive impacts on intention to use AI in health care, while perceived risk had a significant negative impact (B=−0.041, 95% CI −0.066 to −0.015; *P*=.002). In addition, nurses from a secondary hospital or lower (B=−0.273, 95% CI −0.504 to −0.043; *P*=.02) had weaker intention to use AI. Nurses with a master’s degree or higher (B=0.942, 95% CI 0.299-1.585; *P*=.004) and those with positive views on the prospects of AI (B=0.390, 95% CI 0.136-0.644, *P*=.003) had stronger intention to use AI. Overall, compared to the physicians, the nurses had stronger intention to use medical AI (B=0.273, 95% CI 0.055-0.491; *P*=.01).

### Mediating Effects of Performance Expectancy and Effort Expectancy

**Figure 3:**
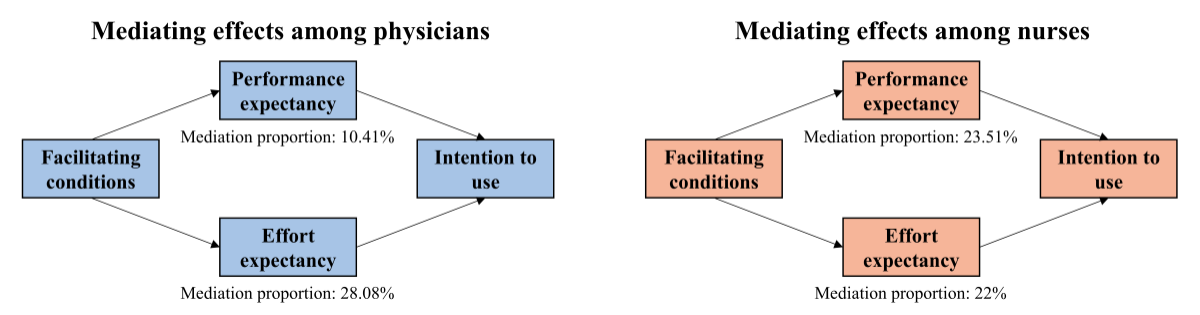
Mediating effects of performance expectancy and effort expectancy among physicians and nurses.

The results of the Karlson-Holm-Breen mediation analysis are presented in Table 5 and Figure 3. We decomposed the total effect of facilitating conditions on intention to use into direct and indirect effects. We then further calculated the proportion of the total indirect effect explained by each mediator. Specifically, for the physicians, the indirect effect of performance expectancy on intention to use accounted for 10.41% of the total effect, while the indirect effect of effort expectancy accounted for 28.08% of the total effect. For the nurses, the indirect effect of performance expectancy on intention to use accounted for 23.51% of the total effect, and the indirect effect of effort expectancy accounted for 22% of the total effect. These findings indicate that performance expectancy and effort expectancy play significant mediating roles between facilitating conditions and intention to use AI among medical staff.

**Table 5:** The mediating effects of performance expectancy and effort expectancy.

Intention to use	Total (n=2705)	Physicians (n=991)	Nurses (n=1714)
	B (95% CI)^a^	B (95% CI)^a^	B (95% CI)^a^
Total effect	0.242 (0.201-0.284)	0.159 (0.092-0.226)	0.291 (0.238-0.343)
Direct effect	0.137 (0.095-0.179)	0.098 (0.031-0.165)	0.158 (0.105-0.212)
Indirect effect	0.105 (0.081-0.129)	0.061 (0.022-0.100)	0.132 (0.101-0.163)

^a^The model was adjusted for demographic information, knowledge of artificial intelligence (AI), experience using AI, hospital attention to medical AI, and personal views on the prospects of medical AI.

### Subgroup Analyses

Subgroup analyses were conducted based on participants’ clinical departments (Multimedia Appendix 6). Among respondents from internal medicine, surgery, and medical technology departments, performance expectancy, effort expectancy, social influence, and facilitating conditions were all significant predictors of intention to use medical AI, consistent with the overall model results. For participants from other departments, performance expectancy, effort expectancy, and facilitating conditions remained significant, whereas social influence was not. This may be attributed to the relatively small sample size in this subgroup. Moreover, a subgroup analysis based on participants’ prior familiarity with medical AI (Multimedia Appendix 7) showed that the key predictors of intention to use were consistent across both subgroups, namely those who had heard of medical AI and those who had not. This indicates that the findings were robust across different levels of baseline awareness.

### Sensitivity Analysis

In the sensitivity analysis, we comprehensively considered the variations in the number of medical staff across diverse geographic regions. We weighted the samples in accordance with the nationwide regional distribution of physicians and nurses and repeated the data analysis. The results of the analysis performed after weighting were comparable to those obtained without weighting (Multimedia Appendices 8-10). This indicates that the sample effectively captured the authentic attitudes and perceptions of physicians and nurses nationwide concerning their intention to adopt medical AI and its influencing factors, thus demonstrating strong representativeness and generalizability.

## Discussion

### Principal Findings

We conducted a cross-sectional survey to investigate the intention of physicians and nurses to adopt medical AI and its influencing factors across 31 provincial-level administrative divisions in mainland China. The results showed that 72.75% (1968/2705) of the participants had a positive intention to use medical AI, while the actual use rate was approximately 19.11% (517/2705). This reflects the open attitude of medical staff toward integrating medical AI into their daily work in the future and highlights the significant gap between medical AI technological innovation and its clinical application. Medical staff who had used AI in health care generally provided favorable evaluations of AI tools but commonly believed that improvements were needed to the stability, reliability, usability, and data privacy of AI systems. Further analysis revealed that performance expectancy, effort expectancy, social influence, and facilitating conditions had significant positive effects on physicians’ and nurses’ intention to use medical AI, while perceived risk had a significant negative impact on nurses’ willingness. Performance expectancy and effort expectancy partially mediated the relationship between facilitating conditions and intention to use. Physicians aged <45 years, working in tertiary hospitals, with <20 years of work experience, and holding an optimistic view of AI’s prospects showed a stronger acceptance of AI. Nurses from tertiary hospitals, with a master’s degree or higher, and holding a positive view of AI’s prospects also demonstrated a stronger intention to adopt medical AI.

### Medical Staff’s Views on Medical AI

In recent years, robust policies implemented by the Chinese government to develop medical AI have effectively promoted its application in disease screening, diagnosis, treatment, and prognostic prediction [37]. Concurrently, the transformative impact of AI has sparked debates within the medical community regarding its benefits and risks [38]. Before policy implementation, it is crucial to investigate the awareness, understanding, and acceptance of medical AI among medical staff, as well as the facilitators and barriers to its adoption. This survey reveals that AI technology holds significant potential; yet, its clinical application remains relatively slow. Of the 2705 participants, 2194 (>80%) viewed medical AI optimistically, with 72% (714/991) to 81.1% (804/991) of the physicians and 73.16% (1254/1714) to 79.93% (1370/1714) of the nurses expressing their intention to use it, despite actual use rates hovering at approximately 19.11% (517/2705). Similarly, the rate of AI use among interventional cardiologists in the United States was 22.1% [39]. Recent multinational surveys reported that approximately 40% of researchers and urologists had used AI chatbots [10,40]. A survey of 288 nurses in Turkey found that 67% believed that using AI would contribute to their professional development, and 27.8% used AI programs [41]. The awareness and application of AI are closely tied to its development level in various professional fields. Given AI’s maturity in radiology, radiologists have significantly more opportunities to use AI for image interpretation. Surveys among radiologists in countries such as the United States, France, and South Korea show acceptance rates ranging from 52.1% to 84.4%, with use rates between 44.9% and 72% [42-47]. Cross-national comparisons of health care AI acceptance and implementation are detailed in Multimedia Appendix 11. Overall, physicians and nurses possess sufficient knowledge and positive attitudes toward AI, but practical application is lacking. This discrepancy can be addressed through increased research investment, improved infrastructure support, and targeted professional training programs [48].

In the health care sector, the safe, effective, and equitable use of AI technology is particularly crucial [1]. Our study revealed that nearly half of the medical staff (1320/2705, 48.8%) had concerns about the ethical safety, accuracy, interpretability, and fairness of AI. While 63.6% (329/517) of the professionals were satisfied with their experience using AI in health care, >80% (435/517) believed that improvements were needed to the reliability and stability of medical AI systems. Addressing concerns regarding the accuracy, reliability, and medicolegal implications of AI is paramount [49,50] because many physicians have expressed expectations for enhanced AI performance and accuracy [40,43,51]. Compared to concerns about ethics, accuracy, interpretability, and fairness, only approximately 28.17% (762/2705) of the medical staff worried that their jobs would be replaced by medical AI. Similar findings were reported in a survey of 487 pathologists from 54 countries: 77% of the respondents held a positive view of medical AI, and only 17.6% were concerned about job displacement [52]. A study involving 1032 Italian radiologists showed that 77% were positive about AI implementation, with only 11.1% expressing concerns about job displacement [53]. Health care professionals in different occupations have varying levels of concern about job displacement [54]. In summary, compared to other risks, concern about job displacement is the least pronounced, further highlighting that AI technology remains in an early stage of development and that there is considerable scope for enhancing its performance relative to human capabilities.

### Factors Influencing the Adoption of AI

The UTAUT model explains users’ acceptance of technology. In 2012, the UTAUT2 model was introduced, incorporating new variables such as hedonic motivation, price value, and habit. However, UTAUT2 is primarily suited to consumer behavior contexts and is not appropriate for explaining medical staff’s acceptance of medical AI in this study. Therefore, we used the original UTAUT model [55]. Our study found that performance expectancy, effort expectancy, social influence, and facilitating conditions positively influenced physicians’ and nurses’ intention to use medical AI, consistent with numerous domestic and international studies [56]. Moreover, perceived risk had no significant impact on physicians’ intention to use medical AI but significantly negatively affected nurses’ intention. This discrepancy may stem from differences in clinical roles and use contexts. Physicians primarily apply AI in assistive diagnosis, documentation, and research, where the perceived risks are more abstract and less likely to affect immediate patient outcomes. By contrast, nurses are often involved in direct patient care and monitoring, where AI errors could have more tangible and immediate consequences. Furthermore, research shows that greater AI knowledge reduces fear of new technology [57,58]. Physicians reported higher levels of AI exposure, use, and education, which may explain why, despite perceiving higher risks, they still have a stronger intention to accept medical AI [59,60]. These insights support tailored strategies for promoting medical AI adoption across clinical roles. For nurses, perceived risk poses a more significant barrier, suggesting the need for targeted training, clear protocols, and institutional support to improve confidence and risk tolerance [61,62]. For physicians, optimizing AI performance, reliability, and clinical integration may be more effective in boosting adoption.

The intention to use AI tools, such as smartwatches, wearable smart devices, and AI chatbots, is primarily influenced by users’ individual conditions. However, the application of medical AI systems first requires medical institutions to have the necessary infrastructure, along with the required knowledge and skills. The impact of facilitating conditions on medical staff’s intention to use AI cannot be overlooked. A survey of 370 members of the Korean Radiological Society showed that 75.5% of the respondents did not use AI tools because their institutions had not purchased them [51]. A survey of 200 members of the World Society of Emergency Surgery revealed that 126 respondents lacked robotic systems in their institutions, which was a major barrier to AI application [63]. Radiologists and technologists in Jordan reported barriers to learning about AI, with the most significant being the lack of expert guidance and support, followed by insufficient funding and investment in new technology [64]. The influence of facilitating conditions on intention to use can be partially explained by performance expectancy and effort expectancy [65]. The presence of facilitating conditions can enhance users’ confidence and self-efficacy in using new technology. When resources for using new technology are readily available, users are more likely to perceive its benefits and have the confidence to overcome difficulties related to its use [66]. Our study results also support this theory because the participants’ performance expectancy and effort expectancy played significant mediating roles between facilitating conditions and intention to use AI in health care.

Our study also revealed that participants aged <45 years, working in tertiary hospitals, holding a master’s degree or higher, and optimistic about medical AI’s prospects, had a stronger intention to use it. Older individuals may be less adaptable to digital health innovations and thus show less acceptance of medical AI than their younger counterparts [67]. This suggests that medical AI development should include enhanced training and guidance for older physicians, with tailored solutions addressing age-specific challenges [68]. Tertiary hospitals exhibit higher attention to medical AI, boasting more abundant AI equipment and technical support compared to secondary hospitals and lower. Consequently, medical staff in tertiary hospitals can access, understand, and use AI earlier, supported by greater social influence and facilitating conditions, resulting in greater intention to use AI. Many primary health care professionals are interested in medical AI but lack exposure opportunities [69]. A significant mismatch exists between their understanding of AI, their preparedness for AI technology, and their desire to learn [70]. To reduce disparities in medical standards across hospital levels, AI projects could be piloted in advanced hospitals, accompanied by increased training and support for medical AI in primary hospitals. Medical staff with higher educational levels are more receptive to AI, likely due to their solid theoretical and practical foundation, which prepares them well for AI acceptance. Prospectively, integrating AI into the medical student curriculum to teach basic AI principles and medical applications could provide students with ample knowledge and technical literacy [71-73]. An optimistic attitude toward AI reflects an individual’s positive belief in its technology, functionality, and efficiency, serving as a measure of new technology-oriented personality traits [74,75]. Optimistic users tend to have lower expectations of potential difficulties and are more likely to believe that using new technology will yield positive outcomes, encouraging them to try these technologies [76]. Demonstrations, training, and publicity can foster an optimistic view of AI among health care professionals, reducing anxiety and fear of unknown technologies [77].

### Implications for Clinical Practice

The findings of this study offer valuable insights into promoting medical AI application in clinical practice and can assist relevant institutions in formulating scientific, rational measures to foster a conducive environment for medical AI development. Regarding performance expectancy, AI developers should design tools that genuinely meet clinical needs, and medical institutions should provide sufficient technical training and equipment support to help medical staff understand the potential benefits of medical AI and learn how to use it effectively, thereby enhancing their confidence in AI’s ability to improve health care quality [78]. For effort expectancy, simplifying medical AI operation processes, providing user-friendly interfaces, and offering timely technical support can enable medical staff to use medical AI more conveniently. Concerning social influence, creating a supportive working atmosphere and learning environment, organizing internal experience-sharing sessions, and inviting successful medical AI users to share their experiences can stimulate other medical staff’s interest and confidence, thereby increasing their intention to use medical AI. Regarding facilitating conditions, governments, manufacturers, and medical institutions should collaborate to provide ample policy, technical, and equipment support, along with legal safeguards. Medical schools could integrate AI skills training into the medical curriculum to equip students with the foundational knowledge and skills necessary for AI use. To address AI risks in health care, AI researchers and developers urgently need to improve AI’s accuracy, transparency, and interpretability. Governments should formulate relevant laws and guidelines, and medical institutions should strengthen data security and privacy protection measures. Through collective efforts, medical staff’s concerns about medical AI can be alleviated [40,79]. These comprehensive measures can effectively enhance medical staff’s intention to use AI, promote the deep integration of medical AI into clinical practice, and inject new vitality into the medical industry’s development.

### Strengths and Limitations

This study involved physicians and nurses from diverse geographic regions in mainland China, with a large sample size ensuring good representativeness. The analysis covered multiple dimensions, including demographic characteristics, AI attitudes, experience of using AI, hospital attention to medical AI, and personal views on AI prospects. This comprehensive approach thoroughly explored physicians’ and nurses’ current perceptions and attitudes toward medical AI, as well as the factors influencing their willingness to use it. The survey questionnaire was designed based on the validated UTAUT model, ensuring the scientific nature of this study. Sensitivity analysis was conducted using weighted adjustments to account for regional differences in the number of physicians and nurses, enhancing the generalizability of the findings by more accurately reflecting the nationwide situation of medical staff.

However, the study has some limitations. First, despite the large sample size, the use of convenience sampling may have introduced selection bias because health care professionals with strong opinions about AI or those more active on social media were more likely to participate. In addition, because the survey was distributed openly through online channels, it was not feasible to track the total number of health care workers who were invited; therefore, an accurate estimation of the response rate could not be obtained. Second, the cross-sectional design of the survey captured data at a single point in time, which limits the ability to establish causal relationships. As all variables were measured simultaneously, any causal interpretations, including those derived from the mediation analysis, should be made with caution. Future studies could adopt a longitudinal design, combining multiple methods such as interviews, observations, and experiments, to validate the results. Third, this preliminary investigation of physicians’ and nurses’ intention to use general-purpose medical AI does not precisely reflect the views of medical staff in different specialties regarding specific AI tools. Given the vast size of China’s medical staff, achieving balanced representation across specialties was challenging, resulting in more than half of the participants (1585/2705, 59%) being from internal medicine departments, which may reduce the generalizability of the results among all health care professionals nationwide. Future research could build on this to conduct more detailed investigations, such as focusing on emergency department nurses’ attitudes toward AI-assisted patient monitoring systems, to address practical clinical problems more effectively. Finally, while the UTAUT model has high explanatory power, it may not cover all influencing factors. Future research could incorporate other theoretical models to more comprehensively explain the factors affecting medical staff’s intention to use medical AI.

### Conclusions

The rapid integration of AI into clinical practice necessitates a systematic investigation into health care professionals’ perspectives on medical AI. Using a nationwide cross-sectional survey of Chinese medical staff, this study identified critical determinants influencing AI adoption in clinical settings. The results indicate that most medical staff hold a positive yet cautious attitude toward medical AI. Although approximately 78.15% (2114/2705) of the respondents expressed willingness to implement medical AI, actual use rate was only about 19.11% (517/2705), highlighting a significant intention-behavior gap. Performance expectancy, effort expectancy, social influence, and facilitating conditions are key factors promoting the intention of physicians and nurses to use AI, while perceived risk significantly negatively impacts nurses’ willingness to adopt it. The effect of facilitating conditions on intention to use can be partially mediated by performance expectancy and effort expectancy. Medical staff are more inclined to adopt medical AI when they believe it enhances work performance, is easy to operate, receives environmental support, and offers favorable conditions. Conversely, their intention to use decreases when they perceive risks related to AI’s accuracy, safety, fairness, transparency, and interpretability. Collaboration among medical AI researchers, developers, government departments, medical institutions, and medical schools is essential. These stakeholders should focus on addressing barriers and leveraging facilitators to promote AI in health care. They should also develop strategies such as improving AI performance, enhancing risk prevention and control mechanisms, increasing technical and equipment support, and fostering a positive environment to alleviate anxiety during the transition to medical AI and ensure its safe and effective application.

## Appendix

Multimedia Appendix 1 The theoretical framework of the modified unified theory of acceptance and use of technology (UTAUT).

Multimedia Appendix 2 Survey questionnaire on intention to use medical artificial intelligence among Chinese physicians and nurses.

Multimedia Appendix 3 Reliability and convergent validity of the questionnaire items.

Multimedia Appendix 4 Discriminant validity of the questionnaire items.

Multimedia Appendix 5 Factors associated with intention to use medical artificial intelligence.

Multimedia Appendix 6 Subgroup analysis of intention to use medical artificial intelligence across different clinical departments.

Multimedia Appendix 7 Subgroup analysis of intention to use medical artificial intelligence based on participants’ awareness of medical artificial intelligence.

Multimedia Appendix 8 Factors associated with intention to use medical artificial intelligence after weighted processing.

Multimedia Appendix 9 Multiple linear regression model for estimating intention to use medical artificial intelligence after weighted processing.

Multimedia Appendix 10 The mediating effects of performance expectancy and effort expectancy after weighted processing.

Multimedia Appendix 11 Cross-national comparisons of medical artificial intelligence acceptance and use.

## Abbreviations

AI: artificial intelligence
STROBE: Strengthening the Reporting of Observational Studies in Epidemiology
TAM: technology acceptance model
TPB: theory of planned behavior
UTAUT: unified theory of acceptance and use of technology
