# Supplementary material for: Attitudes, Perceptions, and Factors Influencing the Adoption of AI in Health Care Among Medical Staff: Nationwide Cross-Sectional Survey Study
Source: J Med Internet Res. 2025 Aug 8;27:e75343. doi: 10.2196/75343 (PMC12374138; doi:10.2196/75343)

# Multimedia Appendix 1. The theoretical framework of the modified UTAUT model. UTAUT: Unified Theory of Acceptance and Use of Technology.


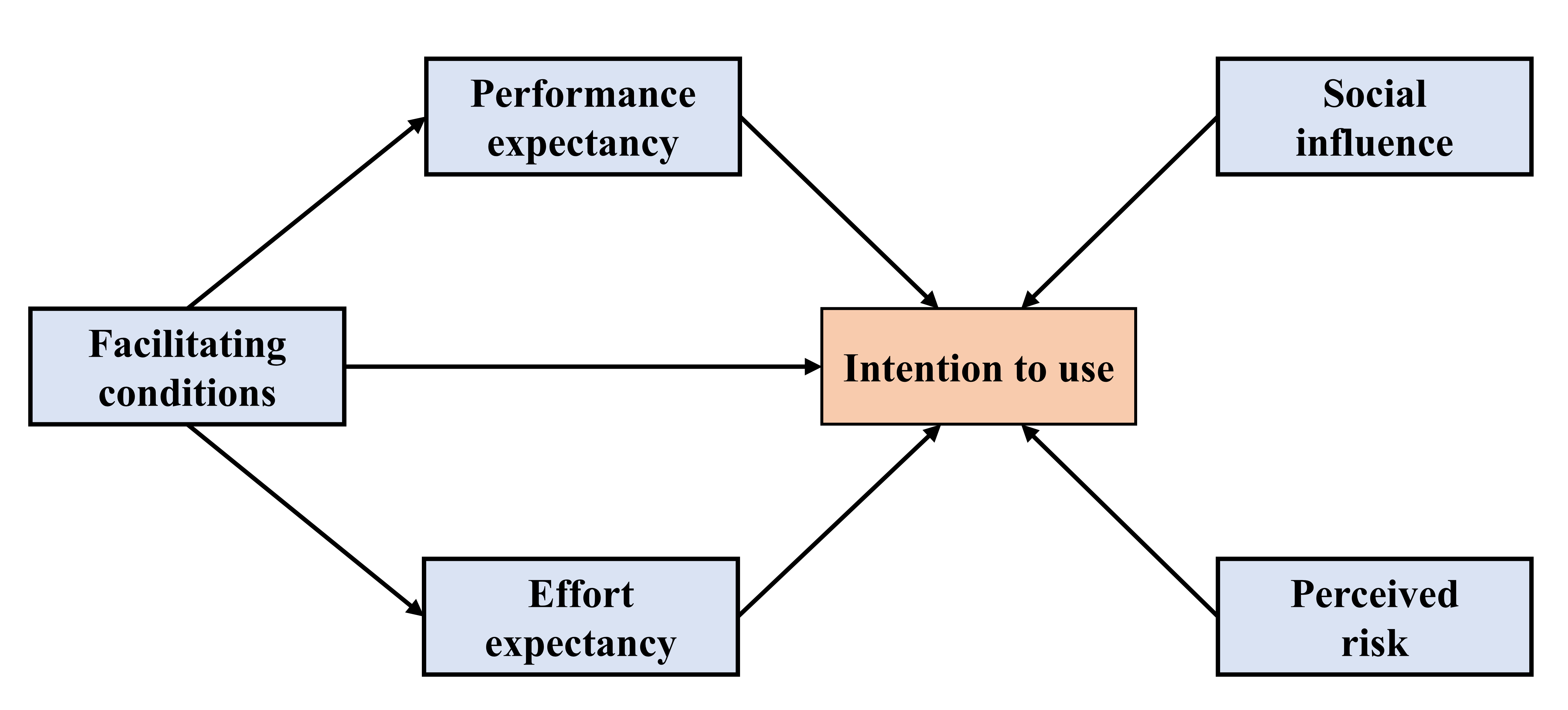

Supplement: Multimedia Appendix 1 [file jmir_v27i1e75343_app1.doc]
