# Supplementary material for: Attitudes, Perceptions, and Factors Influencing the Adoption of AI in Health Care Among Medical Staff: Nationwide Cross-Sectional Survey Study
Source: J Med Internet Res. 2025 Aug 8;27:e75343. doi: 10.2196/75343 (PMC12374138; doi:10.2196/75343)
Supplement: Multimedia Appendix 2 [file jmir_v27i1e75343_app2.doc]

# Multimedia Appendix 2. Survey questionnaire on intention to use medical AI among Chinese doctors and nurses.

Dear medical staff,

Greetings!

With the accelerated progress of technology, AI is gradually integrating into our daily work. The application scope of medical AI technology is continuously expanding, encompassing various fields such as auxiliary diagnosis, individualized treatment, clinical decision support, patient monitoring, disease risk prediction, medical robot operation, medical data management, telemedicine services, as well as teaching and research. In order to gain a deep understanding of your acceptance and intention to use medical AI, we have specially designed this survey questionnaire.

The purpose of this questionnaire is to explore your knowledge, opinions, usage experiences, and perceptions regarding medical AI. We assure you that all the information you provide will be used solely for this study and will be strictly confidential. You have the absolute right to decide whether or not to participate in this study. Additionally, you are free to withdraw from the study at any time during the questionnaire completion process, for any reason or without stating a reason. This decision will have no negative impact on you whatsoever.

It will take approximately 5 minutes to complete this questionnaire. Please answer the following questions truthfully and objectively based on your actual situation. Your responses will greatly contribute to our understanding of the current landscape and future potential of medical AI. We sincerely appreciate your participation and support.

**Part 1: Basic information survey**

1. What is your gender?

£Male

£Female

2. What is your age?

3. What is the province where you reside?

4. What is your highest degree of education?

£Associate’s degree or below

£Bachelor’s degree

£Master’s degree or above

5. What is your occupation?

£Doctor

£Nurse

£Others

6. What is the level of the hospital where you work?

£Tertiary hospital

£Secondary hospital or below

7. In which department do you work?

£Internal medicine department

£Surgery department

£Medical technology department

£Other departments

8. What is your professional title?

£Senior title

£Intermediate title

£Junior title

£No tittle

9. How many years have you been working?

□≤10 years

£11-20 years

□≥21 years

**Part 2: Knowledge and perspectives about medical AI**

1. Have you ever heard of medical AI?

£Yes

£No

2. Have you ever used medical AI?

£Yes

£No

3. What is the level of attention your hospital pays to medical AI?

£Low attention

£General attention

£High attention

4. What is your view on the prospects of medical AI?

£Pessimistic view

£Optimistic view

5. In which areas of application are you more interested in medical AI? (Multiple choices are available)

£Assistive diagnosis

£Personalized treatment

£Clinical decision support

£Patient monitoring

£Disease surveillance

£Medical robotics

£Medical data management

£Telemedicine

£Teaching and research

£Others

**Part 3: Experiences and insights in using medical AI (If you have used medical AI, please continue to answer. If not, please skip this part.)**

1. When did you start using medical AI?

□＜6 months

£0.5-1 years

£1 - 2 years

£2 - 3 years

□＞3 years

2. How satisfied are you with medical AI?

£Very dissatisfied

£Dissatisfied

£Neutral

£Satisfied

£Very satisfied

3. What is the main application for which you use medical AI? (Multiple choices are available)

£Assistive diagnosis

£Personalized treatment

£Clinical decision support

£Patient monitoring

£Disease surveillance

£Medical robotics

£Medical data management

£Telemedicine

£Teaching and research

£Others

4. What aspects do you think need to be improved in the use of medical AI? (Multiple choices are available)

£Improve usability

£Enhance system stability and reliability

£Increase system response speed and processing power

£Improve cost-effectiveness

£Strengthen data security and privacy protection

£Enhance patient interaction features

£Offer more personalized service options

£Strengthen integration with existing medical workflows

£Provide more training and technical support

£Others

**Part 4: Perceptions and intention to use about medical AI (Adoption of the Likert 5 level: from “****Strongly disagree” to “Strongly agree”, assigning values from “1 to 5”)**

1. I think the application of medical AI can enhance my professional skills.

£Strongly disagree £Disagree £Neutral £Agree £Strongly agree

2. I think the application of medical AI can improve my work efficiency.

£Strongly disagree £Disagree £Neutral £Agree £Strongly agree

3. I think the application of medical AI can alleviate my work-related stress.

£Strongly disagree £Disagree £Neutral £Agree £Strongly agree

4. I think the application of medical AI can improve the healthcare experience and enhance patient satisfaction.

£Strongly disagree £Disagree £Neutral £Agree £Strongly agree

5. I think I can easily master the use of medical AI.

£Strongly disagree £Disagree £Neutral £Agree £Strongly agree

6. I think through institutional training, organizational guidance, or assistance from others, I can master the use of medical AI.

£Strongly disagree £Disagree £Neutral £Agree £Strongly agree

7. My colleagues or peers are using medical AI.

£Strongly disagree £Disagree £Neutral £Agree £Strongly agree

8. My organization, colleagues or peers recommend that I use medical AI.

£Strongly disagree £Disagree £Neutral £Agree £Strongly agree

9. Patients themselves hope to utilize medical AI services or products.

£Strongly disagree £Disagree £Neutral £Agree £Strongly agree

10. My organization can provide the necessary funds, equipment, products, or other resources required for using medical AI.

£Strongly disagree £Disagree £Neutral £Agree £Strongly agree

11. My organization can provide the technical support, training, or guidance needed for using medical AI.

£Strongly disagree £Disagree £Neutral £Agree £Strongly agree

12. When issues arise with medical AI products, developers or the organization can assist me in resolving them promptly.

£Strongly disagree £Disagree £Neutral £Agree £Strongly agree

13. I am concerned that medical AI may provide incorrect information, leading to adverse outcomes.

£Strongly disagree £Disagree £Neutral £Agree £Strongly agree

14. I am concerned that medical AI may result in medical disputes, making it difficult to determine responsibility.

£Strongly disagree £Disagree £Neutral £Agree £Strongly agree

15. I am concerned that the lack of transparency and explainability in medical AI may negatively impact the medical process.

£Strongly disagree £Disagree £Neutral £Agree £Strongly agree

16. I am concerned that the use of medical AI may exacerbate inequities in healthcare services.

£Strongly disagree £Disagree £Neutral £Agree £Strongly agree

17. I am concerned that my job might eventually be replaced by medical AI.

£Strongly disagree £Disagree £Neutral £Agree £Strongly agree

18. I am willing to use medical AI to assist me in my work.

£Strongly disagree £Disagree £Neutral £Agree £Strongly agree

19. I am willing to recommend that my colleagues or peers use medical AI to assist in their work.

£Strongly disagree £Disagree £Neutral £Agree £Strongly agree

20. I am willing to recommend that patients use medical AI products or services.

£Strongly disagree £Disagree £Neutral £Agree £Strongly agree

21. I am willing to engage in continuous learning, update my knowledge, and stay informed about the latest developments and technological advancements in the field of medical AI.

£Strongly disagree £Disagree £Neutral £Agree £Strongly agree
