# Supplementary material for: Attitudes, Perceptions, and Factors Influencing the Adoption of AI in Health Care Among Medical Staff: Nationwide Cross-Sectional Survey Study
Source: J Med Internet Res. 2025 Aug 8;27:e75343. doi: 10.2196/75343 (PMC12374138; doi:10.2196/75343)
Supplement: Multimedia Appendix 3 [file jmir_v27i1e75343_app3.doc]

# Multimedia Appendix 3. Reliability and convergent validity of the questionnaire items.

| **Items** | **SFL^a^** | **S.E.^b^** | **Est./S.E.** | ***P value*** | **SMC^c^** | **CR^d^** | **CA^e^** | **AVE^f^** |
| --- | --- | --- | --- | --- | --- | --- | --- | --- |
| **Performance expectancy (PE)** |  |  |  |  |  | **0.933** | **0.931** | **0.776** |
| PE1 | 0.828 | 0.007 | 121.660 | 0.000 | 0.686 |  |  |  |
| PE2 | 0.904 | 0.005 | 200.740 | 0.000 | 0.817 |  |  |  |
| PE3 | 0.898 | 0.005 | 194.300 | 0.000 | 0.807 |  |  |  |
| PE4 | 0.891 | 0.005 | 181.960 | 0.000 | 0.793 |  |  |  |
| **Effort expectancy (EE)** |  |  |  |  |  | **0.912** | **0.907** | **0.838** |
| EE1 | 0.866 | 0.007 | 127.350 | 0.000 | 0.750 |  |  |  |
| EE2 | 0.963 | 0.006 | 174.230 | 0.000 | 0.927 |  |  |  |
| **Social influence (SI)** |  |  |  |  |  | **0.894** | **0.889** | **0.739** |
| SI1 | 0.868 | 0.006 | 143.490 | 0.000 | 0.753 |  |  |  |
| SI2 | 0.930 | 0.005 | 192.640 | 0.000 | 0.864 |  |  |  |
| SI3 | 0.774 | 0.009 | 88.040 | 0.000 | 0.599 |  |  |  |
| **Facilitating conditions (FC)** |  |  |  |  |  | **0.924** | **0.919** | **0.803** |
| FC1 | 0.942 | 0.003 | 274.840 | 0.000 | 0.888 |  |  |  |
| FC2 | 0.954 | 0.003 | 295.900 | 0.000 | 0.910 |  |  |  |
| FC3 | 0.781 | 0.008 | 97.090 | 0.000 | 0.610 |  |  |  |
| **Perceived risks (PR)** |  |  |  |  |  | **0.912** | **0.895** | **0.678** |
| PR1 | 0.868 | 0.006 | 148.300 | 0.000 | 0.754 |  |  |  |
| PR2 | 0.893 | 0.005 | 173.890 | 0.000 | 0.797 |  |  |  |
| PR3 | 0.907 | 0.005 | 189.920 | 0.000 | 0.823 |  |  |  |
| PR4 | 0.774 | 0.009 | 89.470 | 0.000 | 0.599 |  |  |  |
| PR5 | 0.645 | 0.014 | 38.100 | 0.000 | 0.416 |  |  |  |
| **Intention to use (IU)** |  |  |  |  |  | **0.965** | **0.965** | **0.873** |
| IU1 | 0.946 | 0.003 | 373.200 | 0.000 | 0.895 |  |  |  |
| IU2 | 0.962 | 0.002 | 476.860 | 0.000 | 0.926 |  |  |  |
| IU3 | 0.940 | 0.003 | 350.600 | 0.000 | 0.884 |  |  |  |
| IU4 | 0.888 | 0.004 | 197.830 | 0.000 | 0.789 |  |  |  |

^a^SFL: standardized factor loadings.

^b^S.E.: standard error.

^c^SMC: square multiple correlation.

^d^CR: composite reliability.

^e^CA: Cronbach’s alpha.

^f^AVE: average variance extracted.
