# Supplementary material for: Attitudes, Perceptions, and Factors Influencing the Adoption of AI in Health Care Among Medical Staff: Nationwide Cross-Sectional Survey Study
Source: J Med Internet Res. 2025 Aug 8;27:e75343. doi: 10.2196/75343 (PMC12374138; doi:10.2196/75343)
Supplement: Multimedia Appendix 4 [file jmir_v27i1e75343_app4.doc]

# Multimedia Appendix 4. Discriminant validity of the questionnaire items.

|  | Performance expectancy | Effort expectancy | Social influence | Facilitating conditions | Perceived risks | Intention to use |
| --- | --- | --- | --- | --- | --- | --- |
| Performance expectancy | **0.881** |  |  |  |  |  |
| Effort expectancy | 0.732 | **0.915** |  |  |  |  |
| Social influence | 0.437 | 0.445 | **0.860** |  |  |  |
| Facilitating conditions | 0.373 | 0.412 | 0.706 | **0.896** |  |  |
| Perceived risks | -0.038 | 0.014 | -0.008 | 0.011 | **0.823** |  |
| Intention to use | 0.616 | 0.633 | 0.513 | 0.457 | -0.032 | **0.934** |
