# Supplementary material for: Attitudes, Perceptions, and Factors Influencing the Adoption of AI in Health Care Among Medical Staff: Nationwide Cross-Sectional Survey Study
Source: J Med Internet Res. 2025 Aug 8;27:e75343. doi: 10.2196/75343 (PMC12374138; doi:10.2196/75343)
Supplement: Multimedia Appendix 5 [file jmir_v27i1e75343_app5.doc]

# Multimedia Appendix 5. Factors associated with medical AI intention to use (N=2705).

| **Items** | **Doctor (N=991)** | | **Nurse (N=1714)** | |
| --- | --- | --- | --- | --- |
|  | **β (95%CI)** | ***P* value** | **β (95%CI)** | ***P* value** |
| **Gender (Ref. Male)** |  | | | |
| Female | -0.235 (-0.571 - 0.102) |  | -0.13 (-0.480 - 0.221) |  |
| **Age (Ref. <30 years)** |  | | | |
| 30-44 years | -0.113 (-0.757 - 0.531) |  | -0.093 (-0.510 - 0.325) |  |
| ≥45 years | -0.67 (-1.615 - 0.275) |  | 0.2 (-0.455 - 0.856) |  |
| **Region (Ref. North China)** |  | | | |
| Northeast China | 0.038 (-0.513 - 0.590) |  | -0.13 (-0.555 - 0.294) |  |
| East China | **0.54 (0.098 - 0.997)** | **<0.05** | 0.166 (-0.138 - 0.470) |  |
| Central South China | 0.072 (-0.380 - 0.524) |  | 0.047 (-0.352 - 0.445) |  |
| Southwest China | 0.209 (-0.547 - 0.966) |  | -0.244 (-0.654 - 0.165) |  |
| Northwest China | -0.198 (-0.869 - 0.474) |  | -0.307 (-0.853 - 0.239) |  |
| **Educational level (Ref. Associate degree or below)** | | | | |
| Bachelor’s degree | -0.719 (-1.879 - 0.441) |  | 0.024 (-0.326 - 0.373) |  |
| Master’s degree or above | -0.179 (-1.382 - 1.023) |  | 0.664 (-0.142 - 1.469) |  |
| **Hospital grade (Ref. Tertiary hospital)** | | | | |
| Secondary hospital or below | -0.359 (-0.900 - 0.183) |  | **-0.497 (-0.786 - -0.208)** | **<0.05** |
| **Department (Ref. Internal medicine department)** | | | | |
| Surgery department | 0.268 (-0.145 - 0.682) |  | -0.138 (-0.455 - 0.179) |  |
| Medical technology department | -0.345 (-0.930 - 0.240) |  | -0.109 (-0.449 - 0.231) |  |
| Other departments | -0.628 (-1.271 - 0.016) |  | -0.058 (-0.457 - 0.342) |  |
| **Professional title (Ref. Senior title)** | | | | |
| Intermediate title | 0.187 (-0.273 - 0.648) |  | -0.216 (-0.621 - 0.188) |  |
| Junior title | 0.217 (-0.478 - 0.912) |  | -0.285 (-0.795 - 0.226) |  |
| No tittle | -0.415 (-1.624 - 0.794) |  | -0.746 (-1.740 - 0.247) |  |
| **Years of work experience (Ref. ≤10 years)** | | | |  |
| 11-20 years | 0.238 (-0.264 - 0.740) |  | 0.192 (-0.197 - 0.581) |  |
| ≥21 years | **0.872 (0.013 - 1.731)** | **<0.05** | 0.094 (-0.484 - 0.672) |  |
| **Everknow (Ref. No)** |  | | | |
| Yes | -0.185 (-0.819 - 0.449) |  | 0.261 (-0.073 - 0.594) |  |
| **Everuse (Ref. No)** |  | | | |
| Yes | **0.492 (0.088 - 0.896)** | **<0.05** | **0.589 (0.262 - 0.916)** | **<0.05** |
| **Institutional Attention (Ref. Low attention)** | | | | |
| General attention | **0.459 (0.063 - 0.856)** | **<0.05** | 0.109 (-0.181 - 0.399) |  |
| High attention | **0.998 (0.561 - 1.435)** | **<0.05** | **1.066 (0.735 - 1.396)** | **<0.05** |
| **View on prospects (Ref. Pessimistic view)** | | | | |
| Optimistic view | **1.914 (1.483 - 2.344)** | **<0.05** | **1.676 (1.376 - 1.977)** | **<0.05** |
