# Supplementary material for: Attitudes, Perceptions, and Factors Influencing the Adoption of AI in Health Care Among Medical Staff: Nationwide Cross-Sectional Survey Study
Source: J Med Internet Res. 2025 Aug 8;27:e75343. doi: 10.2196/75343 (PMC12374138; doi:10.2196/75343)
Supplement: Multimedia Appendix 6 [file jmir_v27i1e75343_app6.doc]

# Multimedia Appendix 6. The subgroup analysis of intention to use medical AI across different departments (N=2705).

| **Items** | **Internal medical department (N=1585)** | **Surgery department (N=517)** | **Medical technology department (N=356)** | **Other departments (N=247)** |
| --- | --- | --- | --- | --- |
|  | **β (95%CI)** | **β (95%CI)** | **β (95%CI)** | **β (95%CI)** |
| **Performance expectancy** | **0.234 (0.187 - 0.281) ^a^** | **0.231 (0.150 - 0.312) ^a^** | **0.173 (0.082 - 0.263) ^a^** | **0.295 (0.161 - 0.430) ^a^** |
| **Effort expectancy** | **0.569 (0.470 - 0.668) ^a^** | **0.5240.349 - 0.699) ^a^** | **0.440 (0.217 - 0.663) ^a^** | **0.471 (0.184 - 0.758) ^a^** |
| **Social influence** | **0.174 (0.118 - 0.229) ^a^** | **0.124 (0.014 - 0.234) ^a^** | **0.156 (0.009 - 0.303) ^a^** | 0.043 (-0.096 - 0.182) |
| **Facilitating conditions** | **0.131 (0.078 - 0.185) ^a^** | **0.110 (0.008 - 0.212) ^a^** | **0.208 (0.076 - 0.339) ^a^** | **0.197 (0.050 - 0.345) a** |
| **Perceived risks** | -0.022 (-0.048 - 0.004) | -0.023 (-0.072 - 0.026) | 0.021 (-0.044 - 0.086) | -0.026 (-0.100 - 0.047) |
| **Gender (Ref. Male)** |  |  |  |  |
| Female | -0.236 (-0.475 - 0.003) | -0.238 (-0.721 - 0.246) | 0.337 (-0.184 - 0.857) | 0.123 (-0.511 - 0.757) |
| **Age (Ref. <30 years)** |  |  |  |  |
| 30-44 years | -0.044 (-0.378 - 0.290) | -0.592 (-1.278 - 0.094) | 0.247 (-0.709 - 1.203) | 0.659 (-0.229 - 1.546) |
| ≥45 years | -0.149 (-0.692 - 0.395) | -0.267 (-1.306 - 0.771) | 0.338 (-0.945 - 1.621) | 0.917 (-0.395 - 2.229) |
| **Region (Ref. North China)** | | | | |
| Northeast China | -0.142 (-0.497 - 0.214) | 0.005 (-0.563 - 0.573) | -0.116 (-0.849 - 0.617) | -0.241 (-1.119 - 0.638) |
| East China | 0.006 (-0.244 - 0.256) | -0.043 (-0.542 - 0.456) | 0.398 (-0.262 - 1.058) | -0.161 (-0.806 - 0.483) |
| Central South China | -0.14 (-0.438 - 0.159) | -0.231 (-0.761 - 0.299) | 0.528 (-0.187 - 1.243) | 0.624 (-0.164 - 1.412) |
| Southwest China | -0.078 (-0.477 - 0.321) | -0.117 (-0.721 - 0.488) | 0.211 (-0.478 - 0.900) | 0.015 (-1.101 - 1.132) |
| Northwest China | -0.172 (-0.586 - 0.243) | -0.833 (-1.719 - 0.053) | -0.078 (-0.939 - 0.783) | -1.067 (-2.772 - 0.638) |
| **Educational level (Ref. Associate degree or below)** | | | | |
| Bachelor’s degree | 0.296 (-0.026 - 0.618) | -0.328 (-1.104 - 0.447) | 0.105 (-0.706 - 0.915) | -0.266 (-0.990 - 0.458) |
| Master’s degree or above | **0.569 (0.121 - 1.018) ^a^** | -0.22 (-1.139 - 0.699) | -0.094 (-1.293 - 1.105) | 0.366 (-0.867 - 1.599) |
| **Hospital grade (Ref. Tertiary hospital)** | | | | |
| Secondary hospital or below | -0.243 (-0.502 - 0.016) | -0.034 (-0.604 - 0.536) | -0.39 (-0.949 - 0.170) | -0.453 (-1.030 - 0.123) |
| **Professional title (Ref. Senior title)** | | | | |
| Intermediate title | **-0.363 (-0.668 - -0.058) ^a^** | 0.146 (-0.404 - 0.697) | -0.078 (-0.670 - 0.515) | 0.034 (-0.783 - 0.851) |
| Junior title | **-0.486 (-0.889 - -0.084) ^a^** | -0.338 (-1.101 - 0.424) | 0.069 (-0.929 - 1.067) | -0.022 (-1.037 - 0.992) |
| No tittle | -0.587 (-1.339 - 0.165) | -0.34 (-1.761 - 1.081) | -0.001 (-1.705 - 1.703) | -0.185 (-2.368 - 1.998) |
| **Years of work experience (Ref. ≤10 years)** | | | | |
| 11-20 years | -0.128 (-0.424 - 0.167) | 0.535 (-0.006 - 1.075) | 0.044 (-0.798 - 0.887) | 0.284 (-0.484 - 1.052) |
| ≥21 years | -0.215 (-0.706 - 0.276) | 0.332 (-0.530 - 1.194) | 0.41 (-0.760 - 1.580) | 0.174 (-0.944 - 1.292) |
| **Everknow (Ref. No)** |  |  |  |  |
| Yes | -0.083 (-0.382 - 0.217) | -0.302 (-0.880 - 0.275) | 0.41 (-0.332 - 1.153) | 0.289 (-0.395 - 0.972) |
| **Everuse (Ref. No)** |  |  |  |  |
| Yes | 0.14 (-0.125 - 0.406) | 0.175 (-0.286 - 0.636) | 0.218 (-0.387 - 0.823) | -0.198 (-0.938 - 0.542) |
| **Institutional Attention (Ref. Low attention)** | | | | |
| General attention | 0.006 (-0.235 - 0.247) | -0.009 (-0.450 - 0.432) | -0.066 (-0.631 - 0.499) | 0.081 (-0.513 - 0.675) |
| High attention | 0.173 (-0.108 - 0.453) | 0.283 (-0.193 - 0.759) | -0.402 (-1.025 - 0.221) | 0.569 (-0.224 - 1.361) |
| **View on prospects (Ref. Pessimistic view)** | | | | |
| Optimistic view | **0.358 (0.092 - 0.624) ^a^** | **0.638 (0.158 - 1.118) ^a^** | 0.595 (-0.048 - 1.237) | 0.643 (-0.001 - 1.286) |
| **Occupation (Ref. Doctor)** | | | | |
| Nurse | **0.349 (0.047 - 0.652) ^a^** | -0.039 (-0.593 - 0.515) | 0.249 (-0.330 - 0.828) | 0.565 (-0.088 - 1.219) |

^a^P value＜0.05.
