# Supplementary material for: Attitudes, Perceptions, and Factors Influencing the Adoption of AI in Health Care Among Medical Staff: Nationwide Cross-Sectional Survey Study
Source: J Med Internet Res. 2025 Aug 8;27:e75343. doi: 10.2196/75343 (PMC12374138; doi:10.2196/75343)
Supplement: Multimedia Appendix 7 [file jmir_v27i1e75343_app7.doc]

# Multimedia Appendix 7. The subgroup analysis of intention to use medical AI based on awareness of medical AI (N=2705).

| **Items** | **Aware of AI (N=2359) ^a^** | | **Unaware of AI (N=346) ^b^** | |  |
| --- | --- | --- | --- | --- | --- |
|  | **β (95%CI)** | ***P* value** | **β (95%CI)** | ***P* value** |  |
| **Performance expectancy** | **0.211 (0.173 - 0.249)** | **<0.05** | **0.330 (0.232 - 0.429)** | **<0.05** |  |
| **Effort expectancy** | **0.551 (0.468 - 0.633)** | **<0.05** | **0.523 (0.320 - 0.726)** | **<0.05** |  |
| **Social influence** | **0.160 (0.112 - 0.208)** | **<0.05** | **0.130 (0.015 - 0.244)** | **<0.05** |  |
| **Facilitating conditions** | **0.123 (0.079 - 0.168)** | **<0.05** | **0.226 (0.098 - 0.353)** | **<0.05** |  |
| **Perceived risks** | -0.016 (-0.038 - 0.006) |  | -0.04 (-0.101 - 0.022) |  |  |
| **Gender (Ref. Male)** |  |  |  |  |  |
| Female | -0.133 (-0.325 - 0.059) |  | 0.017 (-0.571 - 0.604) |  |  |
| **Age (Ref. <30 years)** |  |  |  |  |  |
| 30-44 years | -0.007 (-0.298 - 0.284) |  | -0.27 (-0.991 - 0.451) |  |  |
| ≥45 years | 0.086 (-0.359 - 0.531) |  | -0.446 (-1.727 - 0.835) |  |  |
| **Region (Ref. North China)** |  |  |  |  |  |
| Northeast China | -0.08 (-0.357 - 0.198) |  | -0.14 (-0.976 - 0.696) |  |  |
| East China | 0.055 (-0.159 - 0.269) |  | 0.066 (-0.462 - 0.593) |  |  |
| Central South China | 0.02 (-0.226 - 0.265) |  | 0.155 (-0.562 - 0.872) |  |  |
| Southwest China | -0.035 (-0.335 - 0.264) |  | 0.226 (-0.627 - 1.078) |  |  |
| Northwest China | -0.282 (-0.625 - 0.061) |  | -0.695 (-2.041 - 0.652) |  |  |
| **Educational level (Ref. Associate degree or below)** | | | | | |
| Bachelor’s degree | 0.075 (-0.216 - 0.366) |  | 0.327 (-0.256 - 0.911) |  |  |
| Master’s degree or above | 0.291 (-0.087 - 0.669) |  | **1.116 (0.035 - 2.196)** | **<0.05** |  |
| **Hospital grade (Ref. Tertiary hospital)** | | | | | |
| Secondary hospital or below | **-0.304 (-0.523 - -0.084)** | **<0.05** | -0.185 (-0.677 - 0.307) |  |  |
| **Department (Ref. Internal medicine department)** | | | | | |
| Surgery department | 0.019 (-0.187 - 0.226) |  | 0.1 (-0.498 - 0.698) |  |  |
| Medical technology department | -0.122 (-0.366 - 0.122) |  | -0.343 (-1.036 - 0.350) |  |  |
| Other departments | 0.043 (-0.248 - 0.334) |  | -0.303 (-0.971 - 0.364) |  |  |
| **Professional title (Ref. Senior title)** | | | | | |
| Intermediate title | -0.13 (-0.369 - 0.108) |  | -0.54 (-1.419 - 0.339) |  |  |
| Junior title | -0.269 (-0.606 - 0.069) |  | -0.696 (-1.661 - 0.268) |  |  |
| No tittle | -0.318 (-0.971 - 0.336) |  | -0.557 (-2.067 - 0.953) |  |  |
| **Years of work experience (Ref. ≤10 years)** | | | | | |
| 11-20 years | 0.041 (-0.212 - 0.293) |  | 0.311 (-0.308 - 0.930) |  |  |
| ≥21 years | 0.036 (-0.363 - 0.434) |  | 0.336 (-0.747 - 1.419) |  |  |
| **Institutional Attention (Ref. Low attention)** | | | | | |
| General attention | -0.04 (-0.243 - 0.163) |  | 0.154 (-0.313 - 0.621) |  |  |
| High attention | 0.184 (-0.044 - 0.412) |  | -0.195 (-0.933 - 0.544) |  |  |
| **View on prospects (Ref. Pessimistic view)** | | | | | |
| Optimistic view | **0.489 (0.262 - 0.715)** | **<0.05** | 0.189 (-0.313 - 0.691) |  |  |
| **Occupation (Ref. Doctor)** |  |  |  |  |  |
| Nurse | **0.294 (0.063 - 0.525)** | **<0.05** | 0.173 (-0.521 - 0.866) |  |  |

^a^Participants who have ever heard of medical AI.

^b^Participants who have never heard of medical AI.
