# Supplementary material for: Attitudes, Perceptions, and Factors Influencing the Adoption of AI in Health Care Among Medical Staff: Nationwide Cross-Sectional Survey Study
Source: J Med Internet Res. 2025 Aug 8;27:e75343. doi: 10.2196/75343 (PMC12374138; doi:10.2196/75343)
Supplement: Multimedia Appendix 8 [file jmir_v27i1e75343_app8.doc]

# Multimedia Appendix 8. Factors associated with medical AI intention to use after weighted processing (N=2705).

| **Items** | **Doctor (N=1242)** | | **Nurse (N=1463)** | |
| --- | --- | --- | --- | --- |
|  | **β (95%CI)** | ***P* value** | **β (95%CI)** | ***P* value** |
| **Gender (Ref. Male)** |  | | | |
| Female | -0.204 (-0.541 - 0.133) |  | -0.063 (-0.398 - 0.272) |  |
| **Age (Ref. <30 years)** |  | | | |
| 30-44 years | -0.178 (-0.814 - 0.458) |  | -0.096 (-0.497 - 0.306) |  |
| ≥45 years | -0.865 (-1.816 - 0.086) |  | 0.143 (-0.518 - 0.804) |  |
| **Region (Ref. North China)** |  | | | |
| Northeast China | 0.037 (-0.689 - 0.764) |  | -0.124 (-0.673 - 0.424) |  |
| East China | **0.550 (0.035 - 1.066)** | **<0.05** | 0.141 (-0.252 - 0.535) |  |
| Central South China | 0.045 (-0.474 - 0.564) |  | 0.036 (-0.362 - 0.433) |  |
| Southwest China | 0.199 (-0.410 - 0.808) |  | -0.282 (-0.732 - 0.169) |  |
| Northwest China | -0.239 (-0.972 - 0.494) |  | -0.33 (-0.862 - 0.203) |  |
| **Educational level (Ref. Associate degree or below)** | | | | |
| Bachelor’s degree | -0.637 (-1.659 - 0.385) |  | 0.089 (-0.246 - 0.425) |  |
| Master’s degree or above | -0.218 (-1.276 - 0.840) |  | **0.944 (0.173 - 1.715)** | **<0.05** |
| **Hospital grade (Ref. Tertiary hospital)** | | | | |
| Secondary hospital or below | -0.468 (-1.014 - 0.079) |  | **-0.413 (-0.710 - -0.116)** | **<0.05** |
| **Department (Ref. Internal medicine department)** | | | | |
| Surgery department | 0.187 (-0.235 - 0.609) |  | **-0.317 (-0.631 - -0.003)** | **<0.05** |
| Medical technology department | -0.425 (-1.009 - 0.158) |  | -0.021 (-0.356 - 0.314) |  |
| Other departments | -0.593 (-1.227 - 0.042) |  | 0.002 (-0.415 - 0.418) |  |
| **Professional title (Ref. Senior title)** | | | | |
| Intermediate title | 0.245 (-0.223 - 0.713) |  | -0.116 (-0.529 - 0.296) |  |
| Junior title | 0.271 (-0.442 - 0.985) |  | -0.238 (-0.759 - 0.282) |  |
| No tittle | -0.741 (-1.873 - 0.391) |  | -0.958 (-1.990 - 0.075) |  |
| **Years of work experience (Ref. ≤10 years)** | | | | |
| 11-20 years | 0.279 (-0.233 - 0.790) |  | -0.01 (-0.387 - 0.367) |  |
| ≥21 years | **1.014 (0.141 - 1.886)** | **<0.05** | -0.079 (-0.669 - 0.510) |  |
| **Everknow (Ref. No)** |  | | | |
| Yes | 0.11 (-0.564 - 0.784) |  | 0.269 (-0.075 - 0.614) |  |
| **Everuse (Ref. No)** |  | | | |
| Yes | **0.452 (0.050 - 0.854)** | **<0.05** | **0.631 (0.321 - 0.940)** | **<0.05** |
| **Institutional Attention (Ref. Low attention)** | | | | |
| General attention | 0.325 (-0.075 - 0.726) |  | 0.015 (-0.276 - 0.306) |  |
| High attention | **0.893 (0.458 - 1.328)** | **<0.05** | **0.999 (0.673 - 1.325)** | **<0.05** |
| **View on prospects (Ref. Pessimistic view)** | | | | |
| Optimistic view | **1.979 (1.545 - 2.414)** | **<0.05** | **1.703 (1.409 - 1.996)** | **<0.05** |
