# Supplementary material for: Attitudes, Perceptions, and Factors Influencing the Adoption of AI in Health Care Among Medical Staff: Nationwide Cross-Sectional Survey Study
Source: J Med Internet Res. 2025 Aug 8;27:e75343. doi: 10.2196/75343 (PMC12374138; doi:10.2196/75343)
Supplement: Multimedia Appendix 9 [file jmir_v27i1e75343_app9.doc]

# Multimedia Appendix 9. Multiple linear regression model for estimating intention to use medical AI after weighted processing (N=2705).

| **Items** | **Total (N=2705)** | | **Doctor (N=1242)** | | **Nurse (N=1463)** | |
| --- | --- | --- | --- | --- | --- | --- |
|  | **β (95%CI)** | ***P* value** | **β (95%CI)** | ***P* value** | **β (95%CI)** | ***P* value** |
| **Performance expectancy** | **0.210 (0.175 - 0.245)** | **<0.05** | **0.144 (0.091 - 0.198)** | **<0.05** | **0.300 (0.252 - 0.347)** | **<0.05** |
| **Effort expectancy** | **0.563 (0.486 - 0.641)** | **<0.05** | **0.638 (0.516 - 0.760)** | **<0.05** | **0.439 (0.338 - 0.541)** | **<0.05** |
| **Social influence** | **0.172 (0.127 - 0.218)** | **<0.05** | **0.255 (0.176 - 0.334)** | **<0.05** | **0.110 (0.055 - 0.165)** | **<0.05** |
| **Facilitating conditions** | **0.113 (0.070 - 0.156)** | **<0.05** | **0.105 (0.035 - 0.174)** | **<0.05** | **0.113 (0.059 - 0.167)** | **<0.05** |
| **Perceived risks** | -0.016 (-0.036 - 0.005) |  | 0.02 (-0.014 - 0.055) |  | **-0.054 (-0.080 - -0.029)** | **<0.05** |
| **Gender (Ref. Male)** | | | | | | |
| Female | -0.126 (-0.302 - 0.050) |  | -0.139 (-0.398 - 0.120) |  | 0.053 (-0.219 - 0.324) |  |
| **Age (Ref. <30 years)** | | | | | | |
| 30-44 years | -0.143 (-0.410 - 0.124) |  | -0.062 (-0.553 - 0.428) |  | -0.212 (-0.534 - 0.111) |  |
| ≥45 years | -0.309 (-0.736 - 0.119) |  | **-1.032 (-1.766 - -0.299)** | **<0.05** | 0.226 (-0.306 - 0.758) |  |
| **Region (Ref. North China)** | | | | | | |
| Northeast China | -0.089 (-0.436 - 0.259) |  | 0.004 (-0.554 - 0.563) |  | -0.125 (-0.566 - 0.317) |  |
| East China | 0.06 (-0.187 - 0.308) |  | 0.193 (-0.206 - 0.591) |  | -0.03 (-0.347 - 0.286) |  |
| Central South China | 0.004 (-0.246 - 0.254) |  | -0.091 (-0.491 - 0.309) |  | 0.101 (-0.220 - 0.421) |  |
| Southwest China | -0.013 (-0.298 - 0.273) |  | -0.055 (-0.524 - 0.414) |  | 0.01 (-0.353 - 0.372) |  |
| Northwest China | -0.308 (-0.652 - 0.035) |  | -0.475 (-1.041 - 0.092) |  | -0.163 (-0.591 - 0.265) |  |
| **Educational level (Ref. Associate degree or below)** | | | | | | |
| Bachelor’s degree | 0.087 (-0.175 - 0.348) |  | -0.494 (-1.280 - 0.292) |  | 0.119 (-0.151 - 0.389) |  |
| Master’s degree or above | 0.238 (-0.100 - 0.576) |  | -0.59 (-1.404 - 0.224) |  | **1.064 (0.441 - 1.686)** | **<0.05** |
| **Hospital grade (Ref. Tertiary hospital)** | | | | | | |
| Secondary hospital or below | **-0.297 (-0.509 - -0.086)** | **<0.05** | **-0.547 (-0.968 - -0.126)** | **<0.05** | -0.206 (-0.446 - 0.033) |  |
| **Department (Ref. Internal medicine department)** | | | | | | |
| Surgery department | -0.02 (-0.217 - 0.177) |  | 0.087 (-0.238 - 0.412) |  | -0.136 (-0.390 - 0.117) |  |
| Medical technology department | -0.123 (-0.357 - 0.112) |  | -0.416 (-0.866 - 0.033) |  | -0.032 (-0.301 - 0.237) |  |
| Other departments | 0.046 (-0.232 - 0.325) |  | -0.28 (-0.769 - 0.209) |  | 0.266 (-0.070 - 0.602) |  |
| **Professional title (Ref. Senior title)** | | | | | | |
| Intermediate title | -0.113 (-0.342 - 0.117) |  | -0.127 (-0.488 - 0.235) |  | -0.127 (-0.459 - 0.206) |  |
| Junior title | -0.292 (-0.611 - 0.028) |  | -0.192 (-0.746 - 0.362) |  | -0.361 (-0.782 - 0.060) |  |
| No tittle | **-0.576 (-1.145 - -0.008)** | **<0.05** | -0.751 (-1.623 - 0.120) |  | -0.402 (-1.235 - 0.430) |  |
| **Years of work experience (Ref. ≤10 years)** | | | | | | |
| 11-20 years | 0.081 (-0.152 - 0.314) |  | 0.149 (-0.244 - 0.543) |  | 0.098 (-0.205 - 0.401) |  |
| ≥21 years | 0.216 (-0.171 - 0.602) |  | **0.898 (0.227 - 1.569)** | **<0.05** | -0.303 (-0.778 - 0.172) |  |
| **Everknow (Ref. No)** | | | | | | |
| Yes | -0.007 (-0.259 - 0.245) |  | -0.028 (-0.550 - 0.493) |  | 0.035 (-0.243 - 0.312) |  |
| **Everuse (Ref. No)** | | | | | | |
| Yes | 0.067 (-0.130 - 0.265) |  | 0.167 (-0.149 - 0.483) |  | 0.066 (-0.189 - 0.322) |  |
| **Institutional Attention (Ref. Low attention)** | | | | | | |
| General attention | -0.055 (-0.243 - 0.133) |  | -0.012 (-0.324 - 0.300) |  | -0.15 (-0.385 - 0.084) |  |
| High attention | 0.136 (-0.078 - 0.350) |  | -0.061 (-0.412 - 0.290) |  | 0.266 (-0.003 - 0.536) |  |
| **View on prospects (Ref. Pessimistic view)** | | | | | | |
| Optimistic view | **0.544 (0.337 - 0.750)** | **<0.05** | **0.563 (0.209 - 0.918)** | **<0.05** | **0.449 (0.199 - 0.699)** | **<0.05** |
| **Occupation (Ref. Doctor)** | | | | | | |
| Nurse | 0.188 (-0.019 - 0.395) |  |  |  |  |  |
