# Supplementary material for: Attitudes, Perceptions, and Factors Influencing the Adoption of AI in Health Care Among Medical Staff: Nationwide Cross-Sectional Survey Study
Source: J Med Internet Res. 2025 Aug 8;27:e75343. doi: 10.2196/75343 (PMC12374138; doi:10.2196/75343)
Supplement: Multimedia Appendix 10 [file jmir_v27i1e75343_app10.doc]

# Multimedia Appendix 10. The mediating effects of performance expectancy and effort expectancy after weighted processing (N=2705).

| **Intention to use** | **Total (N=2705)** | **Doctor (N=1242)** | **Nurse (N=1463)** |
| --- | --- | --- | --- |
|  | **β (95%CI)^a^** | **β (95%CI)^a^** | **β (95%CI)^a^** |
| Total effect | 0.210 (0.168 - 0.252) | 0.166 (0.096 - 0.235) | 0.245 (0.193 - 0.298) |
| Direct effect | 0.113 (0.070 - 0.156) | 0.105 (0.035 - 0.174) | 0.113 (0.059 - 0.167) |
| Indirect effect | 0.097 (0.073 - 0.120) | 0.061 (0.024 - 0.098) | 0.133 (0.101 - 0.164) |
| **Mediators** | **Mediation (%)** | | |
| Performance expectancy | 16.61 | 10.07 | 27.74 |
| Effort expectancy | 29.52 | 26.77 | 26.26 |

^a^ The model was adjusted for demographic information, knowledge of AI, usage experience, hospital attention, and personal views on the prospects of medical AI.
