# Supplementary material for: Attitudes, Perceptions, and Factors Influencing the Adoption of AI in Health Care Among Medical Staff: Nationwide Cross-Sectional Survey Study
Source: J Med Internet Res. 2025 Aug 8;27:e75343. doi: 10.2196/75343 (PMC12374138; doi:10.2196/75343)
Supplement: Multimedia Appendix 11 [file jmir_v27i1e75343_app11.doc]

# Multimedia Appendix 11. Cross-national comparisons of medical AI acceptance and usage.

| **The countries surveyed** | **Participants** | **Number of acceptors/ sample size** | **Acceptance rate** | **Number of users/ sample size** | **Usage rate** | **Reference** |
| --- | --- | --- | --- | --- | --- | --- |
| Globe | Medical researchers | 1428/2048 | 69.7% | 945/2125 | 44.5% | [10] |
| China | Colposcopists | 267/284 | 94.0% | 88/284 | 31.0% | [11] |
| Globe | Gastroenterologists | 357/374 | 95.5% | 25/374 | 6.7% | [12] |
| China | Radiology residents | 2866/3666 | 78.2% | 2639/3666 | 72.0% | [15] |
| America | Interventional cardiologists | 332/521 | 63.7% | 115/521 | 22.1% | [39] |
| Globe | Urologists | 251/322 | 78% | 146/322 | 45.3% | [40] |
| Turkey | Nurses | 193/288 | 67.0% | 80/288 | 27.8% | [41] |
| France | Young radiation oncologists | 94/117 | 80.3% | 71/117 | 60.7% | [42] |
| Korea | Neuroradiologists | 38/73 | 52.1% | 43/73 | 58.9% | [43] |
| Austria | Radiologists | 54/64 | 84.4% | 44/63 | 69.8% | [44] |
| America | Radiologists | 148/211 | 70.1% | 144/211 | 68.2% | [45] |
| Sweden | Breast radiologists | 29/47 | 61.7% | 25/47 | 53.2% | [46] |
| Australia | Medical staff in breast imaging | 46/62 | 74.2% | 40/89 | 44.9% | [47] |
